# Molecular imaging uncovers growth media influence on biofilms’ EPS production

**DOI:** 10.3389/fchem.2025.1703055

**Published:** 2026-02-11

**Authors:** Gabriel D. Parker, Andrew Plymale, Jacqueline Hager, Luke Hanley, Xiao-Ying Yu

**Affiliations:** 1 Department of Chemistry, University of Illinois Chicago, Chicago, IL, United States; 2 Materials Science and Technology Division, Oak Ridge National Laboratory, Oak Ridge, TN, United States; 3 Earth Systems Science Division, Pacific Northwest National Laboratory, Richland, WA, United States

**Keywords:** paenibacillus, biofilms, ToF-SIMS, fatty acid, metabolite, metabolic stress, growth media

## Abstract

Biofilm growth in a laboratory typically requires media rich with amino acids and other nutrients for bacterial metabolism. Microbial biofilms consist of clusters of planktonic cells grouping and secrete extracellular polymeric substance (EPS). The EPS is a complex mixture consisting of polysaccharides, fatty acids, and lipids as well as primary and secondary metabolites among other biomolecules. Choice of growth medium is important to culturing microbes, as it should allow the bacteria to replicate at rapid rates. Herein, we study the effect of media selection on biofilm culture. We investigated three growth media, including two common complex growth media, namely, Luria Broth (LB) and Tryptic Soy Broth (TSB), and a minimal growth medium, hydrogen oxidizing de-nitrifier (HOD). The latter was supplemented with glucose as the carbon energy source for aerobic growth, and nitrate was not incorporated into the media. HOD was developed to cultivate hydrogenotrophic groundwater bacteria isolated from the Hanford Site in Richland, WA. A *Paenibacillus* strain originating in the Hanford Site subsurface was selected as the model biofilm system. We used time-of-flight secondary ion mass spectrometry (ToF-SIMS) to investigate the cultures over a 7-day period. Three time points were chosen based on the bacterial growth curve, corresponding to the log phase, stationary phase, and death phase, respectively. The SIMS spectral and two-dimensional (2D) imaging results show that the fatty acid peaks in HOD-grown biofilms are different from those cultured in the complex media. In the HOD-grown biofilms, biomarkers indicative of bacterial stress are localized as evidenced in ToF-SIMS 2D images. Our SIMS 2D image findings also show that distributions of prominent fatty acids and lipids, as components of the EPS and possibly bacterial plasma membrane, are influenced by the growth medium. HOD, among the three media studied, seems to offer the most distinctive metabolic behavior of the selected biofilm strain. Minimal media, such as HOD, are suggested as a suitable choice to study microbial effects on materials corrosion due to the nature of the minimal medium effects, which offer good insights into the metabolic process of biofilms.

## 1 Introduction

Rhizobacteria are soil bacteria that are contained in the rhizosphere, which is generally defined as the zone of soil under the influence of plant roots. Within the rhizosphere, there are bacterial communities and specific microbial species which enhance plant growth, induce corrosion, and cause pathogenic responses (Chepsergon and Moleleki, 2023; Tkachuk and Zelena, 2022). Common rhizosphere bacteria include *Paenibacillus*, *Pseudomonas*, and *Arthrobacter* strains, and they have been studied over the past 2 decades because of their plant growth promotion properties, and these bacteria are also called plant growth promoting rhizobacteria (PGPR) (Lugtenberg and Kamilova, 2009; Manjunatha et al., 2022; Sanchez-Mahecha et al., 2022; Vater et al., 2017). The *Paenibacillus* genus has been studied recently, and it shows possible microbiologically influenced corrosion of material (Marvasia et al., 2009; Parker et al., 2024). Microbial interactions occur due to the large number of bacterial cells that inhabit the soil (Raynaud and Nunan, 2014). Investigation of specific bacterial strains with plant growth properties or material degradation as a model rhizobacteria, such as *Paenibacillus* genus, is highly desirable for many industries, such as agriculture, biotechnology, waste management, and general medicine (Lebano et al., 2024).

*Paenibacillus* sp. 300A (abbreviated as 300A) was isolated from the subsurface of the 300 Area in the U.S. Department of Energy’s Hanford Site, located in southeastern Washington state. The Hanford Site is an area contaminated with uranium due to past nuclear processing and waste storage (Ahmed et al., 2012). This 300A species is a Gram-positive, rod-shaped facultative anaerobe. It is a motile, swarming bacterium that readily forms biofilms (Berghouse et al., 2025; Perez et al., 2022). Strain 300A was isolated from subsurface sediments that were seasonally saturated with groundwater (Ahmed et al., 2012). Presumably, the strain trickled down into the Hanford vadose zone and groundwater from the plant rooting zone above (Balkwill et al., 1998; Benzine et al., 2013). *Paenibacillus* rhizobacteria species are known for production of biologically relevant compounds, such as nonribosomally formed peptides, polyketides, antibiotics, phytohormones, lytic enzymes, other biocins, and a wide range of exopolysaccharides, which aid in plant growth and antimicrobial effects (Grinev et al., 2020; Vater et al., 2017). Growth media comprised of nutrients such as amino acids, vitamins, and minerals are used to supplement the growth of the bacteria by controlling factors such as pH, water, carbon and nitrogen sources, as well as colony size (Bonnet et al., 2020). Growth media play a vital role in the metabolic production of extracellular polymeric substances (EPS), which contain fatty acids, amino acids, polysaccharides, and lipids (Sanchez-Rosario and Johnson, 2021), as the backbone of the biofilms.

Many analytical tools, such as X-ray photoelectron spectroscopy (Wei et al., 2021), nuclear magnetic resonance microimaging and spectroscopy (Renslow et al., 2017), scanning electron microscopy (McCutcheon and Southam, 2018), confocal laser scanning microscopy, and Raman spectroscopy (Gieroba et al., 2020), have been used to study biofilms. However, these techniques lack the selectivity to resolve larger molecules, such as fatty acids, proteins, and lipids. Mass spectral imaging, such as matrix assisted laser desorption ionization (MALDI) mass spectrometry, desorption electrospray ionization (DESI) mass spectrometry, and time-of-flight secondary ion mass spectrometry (ToF-SIMS), were used for characterization of biological materials, due to their ability to gather spectra information and images. Comparing between MALDI (McCaughey et al., 2022) and SIMS (Hua et al., 2016), ToF-SIMS has advantages to study microbial systems because its lateral/spatial resolution allows analysis within a single cell, while providing adequate mass resolving power and superior surface sensitivity of organics (Fernández-Remolar et al., 2015; Parker et al., 2023; Wei et al., 2017; Wei et al., 2020). It is worth noting that MALDI has excellent mass resolution upwards to 120k, when utilizing an orbitrap (Angerer et al., 2022). Both static SIMS (Zhang et al., 2021; Zhang Y. et al., 2022; Zhang et al., 2023) and *in situ* liquid SIMS (Zhang et al., 2021; Zhang Y. et al., 2022; Zhang et al., 2023) were used to study biofilms. Identifying compounds that bacterial strains secrete via different media is crucial to diverse biofilm applications. Detailed characterization of biofilms also provides physical data towards building a community database for identification of small molecules inherent in EPS and biofilms using ToF-SIMS. Growth time and growth media could affect bacterial secretions observed in the 300A biofilm cultures by ToF-SIMS.

In this work, commonly used rich growth media, namely, Luria Broth (LB) and Tryptic Soy Broth (TSB), and a minimal growth medium (hydrogen oxidizing denitrifier (HOD)) were chosen for comparison of biological secretions and biofilm formation over time. The HOD media was originally formulated to simulate growth conditions of the subsurface groundwater conditions of the Hanford region (Lee et al., 2012; Lee et al., 2015). The HOD minimal media has been used to study the corrosion of materials via microbial induced corrosion recently (Parker et al., 2024). The molecules with relevance to EPS (Gao et al., 2019; Seviour et al., 2019) are the focus of the SIMS spectral analysis. ToF-SIMS spectroscopy and two dimensional (2D) molecular imaging acquired from biofilms are reported. The mid-mass region (*m/z* 200–350) shows detection of metabolites, fatty acids, and fragments of lipids and polysaccharides from the 300A biofilms as discussed in several recent papers (Ding et al., 2019; Wei et al., 2017; Wei et al., 2020; Zhang et al., 2021; Zhang S. et al., 2022). Additionally, our results show that sulfate molecules could possibly originate during the bacterial metabolism processes of the biofilms when cultured using the HOD medium, providing information regarding cellular death and stress responses.

## 2 Materials and methods

### 2.1 Bacterial culture

**FIGURE 1:**
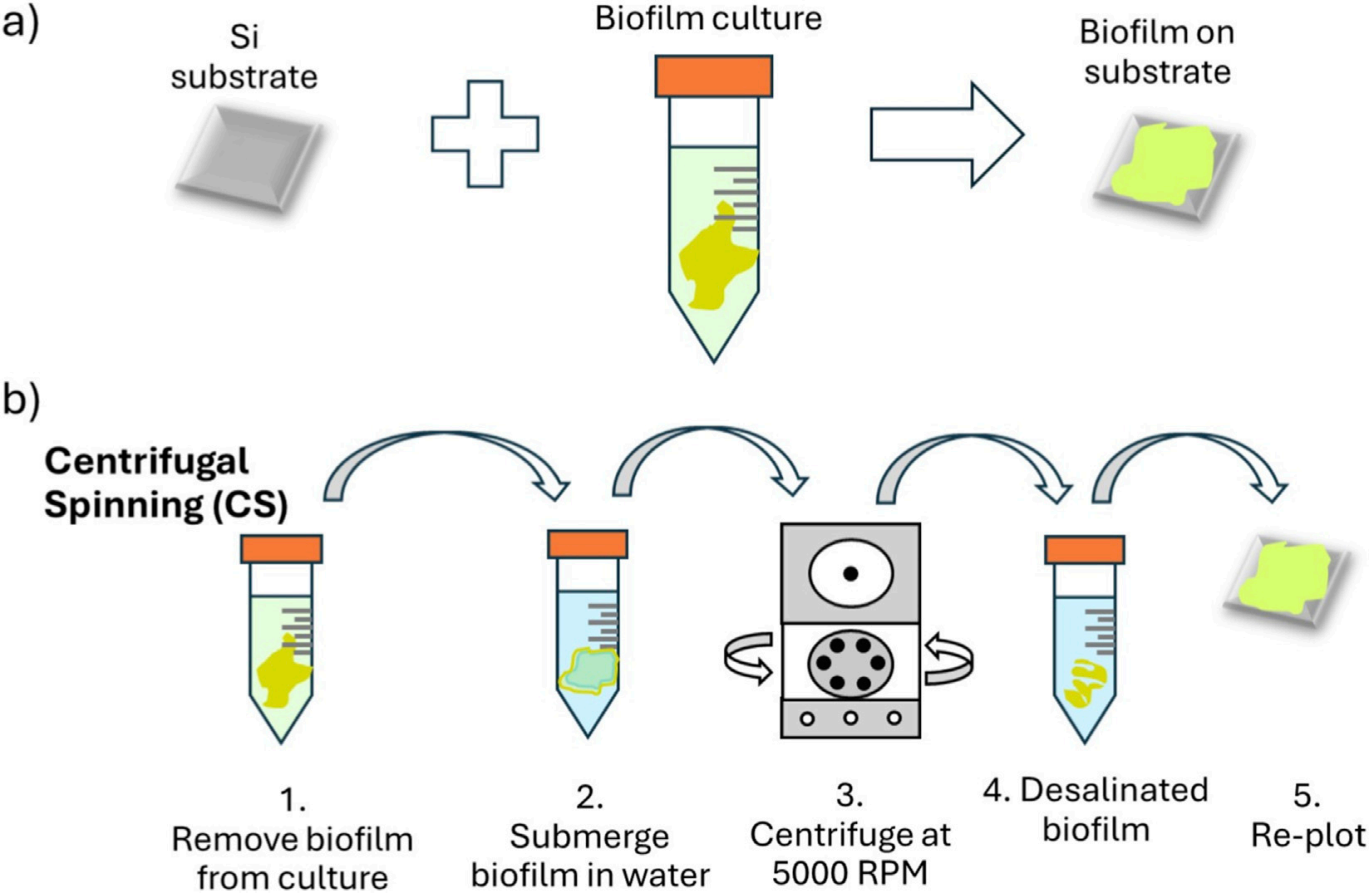
Schematics of the biofilm sample preparation for SIMS analysis: **(a)** substrate selection, culturing, and preparation; and **(b)** the desalination process.

The *Paenibacillus* sp. 300A bacteria strain was cultured on LB, TSB, and HOD agar plates (Zhang et al., 2021; Zhang Y. et al., 2022). The HOD, TSB, and LB media composition can be found within Supplementary Tables S1–S3, respectively. All agar plates were incubated at 30 °C for 24 h. After assessing cultures for contamination, one or two pure colonies were inoculated in 10 mL of medium in 50 mL falcon tubes. Liquid cultures were grown without shaking at 25 °C to ∼0.2 optical density (OD_600_) (Ding et al., 2016; Komorek et al., 2017). The biomass was then harvested by centrifugation for 5 min at 5000 rpm. After centrifugation, the supernatant was discarded and replaced with 1 mL sterile deionized (DI) water and vortexed to resuspend. This process was repeated three times. 200 μL DI water was added for a final resuspension. The biofilms were then recast onto sterilized silicon (Si) wafers and air dried under laminar flow within a biosafety cabinet (BSC). Biofilms were grown for 1-day, 3- days, and 7- days, respectively, and conditions of the biofilms were verified via optical microscope. Figure 1 shows a schematic of the culture and desalination process. The latter refers to the centrifugation of the biofilm. ToF-SIMS spectra of HOD medium control and various peaks which were used for peak area reduction calculations to determine the effect of centrifugal spinning (See Supplementary Figure S1 **)**. Values associated with peak reduction are found in Supplementary Table S4 **.** The centrifugal spinning method reduces media peaks up to 99.6% for biofilm grown in HOD. The dehydrated samples were housed, individually, in a small Petri dish sealed with parafilm within a −20 °C freezer until analysis was performed.

Sterilized Si wafers were prepared by sonication in 30 mL ethanol, isopropanol, and acetone, respectively for 5 min. The wafers were then blown dry with nitrogen gas after each sonication bath. The cleaned Si wafers were then treated with UV-ozone (model No. 342, Jetlight Company Inc.) for 1 min to render the surface hydrophilic (Ding et al., 2016; Zhang et al., 2023).

### 2.2 ToF-SIMS

Dehydrated biofilm samples were loaded into the loadlock chamber first and reached the pressure of 5 × 10^−6^ mbar before loading into the main chamber of the ToF-SIMS instrument. Main chamber pressure was maintained between 3 × 10^−7^ – 5 × 10^−8^ mbar. ToF-SIMS spectra were obtained using an IONTOF TOF-SIMS 5 equipped with a 25 keV Bi_3_^+^ metal ion gun. The static limit is defined as <10^12^ ions/cm^2^ (Dubey et al., 2011; Passarelli and Winograd, 2011; Xu et al., 2004). In this work, the primary ion dose density was 6.9 × 10^11^ ions/cm^2^. Surface spectra were collected below the static limit; therefore, surface damage is insignificant. This static limit defines the threshold before surface damage occurs which could result in sample alteration. Above the limit of 10^12^ ions/cm^2^, the ratio of damaged sample becomes higher than 1% (Sepahyar Lorentzen et al., 2025). The spectral pixel resolution was 128 × 128, and the pulse length 25 ns with the static primary current of 27 nA. Data were collected with 1 pulse/pixel. The raster size for each region of interest was 500 μm × 500 µm. The number of scans was 25. Scans refer to the collection of mass spectra or data points. The electron flood gun was used as needed during the analysis of the biofilm sample. The 21 eV electron flood gun was used for each biofilm sample to reduce charge buildup at the sample surface. The electron flood gun had a filament current of 2.05 A for both positive and negative measurements. The flood gun also had an extraction bias of −30 V for negative mode spectra and −20 V for positive mode spectra. The measurement cycle time was 100 µs, ensuring data collections up to *m/z* 800. Each measurement was performed on a new sample that had not undergone ToF-SIMS experimentation previously to exclude any beam effects from the dataset.

### 2.3 SIMS data analysis

Calibration points for each of the biological samples were selected to be the same when possible. Calibration peaks used for the 300A strain were *m/z* ^*–*^ 14.02 CH_2_^−^, *m/z* ^*–*^ 41.00 CNO^−^, *m/z* ^*–*^ 49.01 C_4_H^−^, *m/z* ^*–*^ 61.01 C_5_H^−^, and *m/z* ^*–*^ 283.26 C_18_H_35_O_2_^−^ in the negative mode; and *m/z* ^*+*^ 15.02 CH_3_^+^, *m/z* ^*+*^ 29.04 C_2_H_5_^+^, *m/z* ^*+*^ 53.04 C_4_H_5_^+^, *m/z* ^*+*^ 67.05 C_5_H_7_^+^, and *m/z* ^*+*^ 83.09 C_6_H_11_^+^ in the positive mode, respectively. Peaks reported within the Tables have a signal to noise ratio (S/N) of 3 or greater. The data were mass calibrated before ASCII conversion. Data plotting was done using OriginPro 2023 after the data was extracted from the SIMS data files and converted to ASCII formate. Media controls were analyzed, and peaks were selected for comparison. Biofilm and media control peak lists were exported into Excel® where the sample values and media values were sorted based on mass values in SurfaceLab. Supplementary Figure S2 provides a visualization of peaks for the illustration of calibration accuracy.

Peak identifications of molecules was obtained using the IONTOF *SurfaceSpectra v7* software that suggested mass formula with a mass deviation of less than 65 ppm and based on the analyst’s knowledge of the biological system supported with literature search. Mass deviation ± 65 ppm is the SIMS community standard as provided by the American Vacuum Society (Graham, 2024). The SIMS mass accuracy is defined as ΔM: = Abs (10^6^ × (*m/z*_obs_ − *m/z*_the_)/*m/z*_the_) (expressed in ppm), where *m/z*_obs_ and *m/z*_the_ refer to the observed and theoretical mass to charge ratio of a specific peak in the negative or positive ion mode (Gilmore and Seah, 2000; Yu et al., 2023). Peak assignment was based on the mass accuracy from the associated peak value to the corresponding value within these databases. Peak assignments were verified by literature search pertaining to the targeted biological molecules. PubMed, ChEBI, LipidMaps, MetabolomicsWorkbench, and KEGG databases were surveyed for molecular assessment. The reported peak identifications are possibilities which are the best match based on relative mass accuracy, biofilm biological functions of the analytes, and knowledge accumulated in published literature. More unambiguous identification of molecules would need to be corroborated with tandem MS and/or other complimentary techniques (Baig et al., 2015).

## 3 Results and discussion

### 3.1 Media comparison show TSB and LB promote fatty acid and lipid identification

*Paenibacillus* sp. 300A strains were cultured over a period of 7 days with three-time stamps to represent exponential growth (log phase), stationary phase, and death phase, based on the bacterial growth curve. Supplementary Figures S3, S4 shows the 300A growth curve when cultured under bulk aerobic conditions using the HOD medium with 10 mM glucose as carbon source. Growth curves of *Paenibacillus* have been reported (Kim et al., 2016). Although Kim *et al.’*s growth curves used a different *Paenibacillus* strain, their results are similar to the HOD grown bacteria reported in this work. Based on the growth curve, the optimal time point for growth is between 35 and 95 h. It is during this period that the biofilm becomes mature and reaches the static growth limit. As time continues, the optical density declines, indicating that the bacteria enter the death phase. From the static time point at 30 h of growth to the entrance of the death phase at 95 h, there is a quantifiable decrease in optical density of 40%. Without supplemental nutrient source, the live cell count in the biofilm continues to decrease as reflected in their optical density. The time points for when there is initial growth, static growth, and a decline of growth are of particular interest because they may provide insights into the molecules produced when the biofilm is just forming, at maturity, and dying.

**FIGURE 2:**
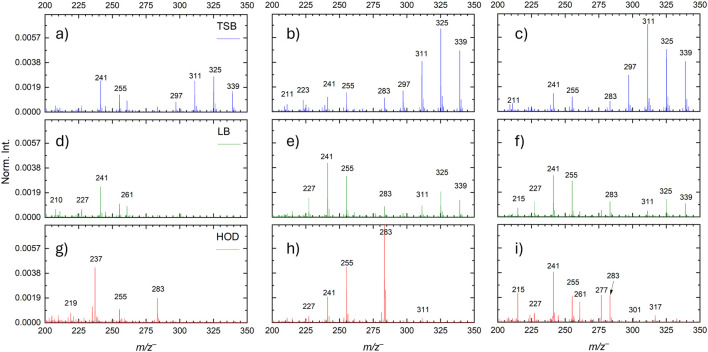
SIMS spectral comparisons of Paenibacillus sp. 300A biofilms grown in TSB, LB, and HOD media for 1–day, 3–days, and 7–days, respectively. **(a–c)** correspond to TSB media growth during 1, 3, and 7–day periods. **(d–f)** correspond to LB media growth during 1, 3, and 7–day periods. **(2g–i)** correspond to HOD media growth during 1, 3, and 7–day periods.

**FIGURE 3:**
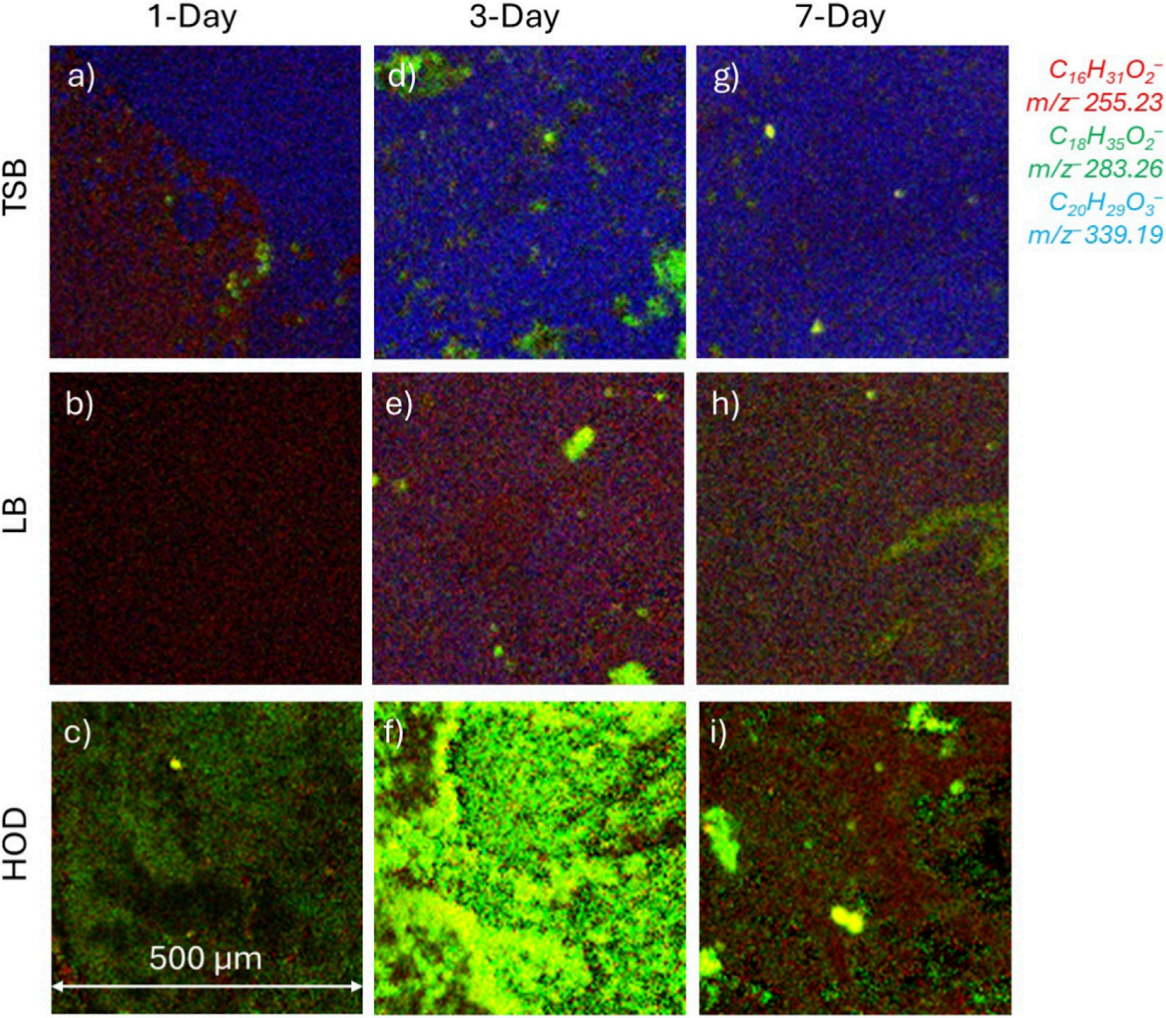
2D SIMS images of red, green, blue (RGB) showing overlays of prominent fatty acids and lipids identified as EPS components and possibly bacterial plasma membrane: *m/z* ^−^ 255.23 C_16_H_31_O_2_^−^ palmitic acid (red), *m/z* ^−^ 283.26 C_18_H_35_O_2_^−^ stearic acid (green), and *m/z* ^−^ 339.19 C_22_H_27_O_3_^−^ a lipid (blue). Images are normalized to total ion values. **(a,d,g)** correspond to TSB media growth during 1, 3, and 7–day periods. **(b,e,h)** correspond to LB media growth during 1, 3, and 7–day periods. **(c,f,i)** correspond to HOD media growth during 1, 3, and 7–day periods.

Figure 2 displays normalized ToF–SIMS spectral comparisons of the three time points, 1-day, 3-day, and 7-day, of the three media chosen to grow the biofilms, namely, TSB, LB, and HOD. The blue color spectra are representative of the TSB, while green and red are LB and HOD, respectively. SIMS spectra have been normalized to the total ion counts for ease of comparisons. Supplementary Figure S5 shows the spectra in absolute counts for comparison. Initially at 1-day growth, the only common peak among the three media is the fatty acid of *m/z* ^*–*^ 255 C_16_H_31_O_2_^−^, palmitic acid, a product from the biofilms. Palmitic acid contributes to the bacteria’s cellular structure, and it is also a product involved in cellular stress responses (Bisht and Chauhan, 2020; Ding et al., 2016; Ory et al., 2023). The spectra are quite different from one another at 1-day growth, which can be attributed to the growth media compositions. Supplementary Figure S6 shows the media controls spectra by ToF-SIMS. The TSB spectra, Figure 3a, shows the highest amount of fatty acid and lipid molecules within the observed region at the log phase among the three media. The peaks *m/z* ^*–*^ 297.14 C_19_H_21_O_3_^−^ or C_12_H_26_O_6_P^−^, *m/z* ^*–*^ 311.16 C_20_H_23_O_3_^−^ or C_13_H_28_O_6_P^−^, *m/z* ^*–*^ 325.18 C_21_H_25_O_3_^−^ or C_14_H_30_O_6_P^−^, and *m/z* ^*–*^ 339.19 C_22_H_27_O_3_^−^ or C_15_H_32_O_6_P^−^, are attributed to lipids, whereas *m/z* ^*–*^ 241.21 C_15_H_29_O_2_^−^ and *m/z* ^*–*^ 255.23 C_16_H_31_O_2_^−^ fatty acids. While the mass deviation of the lipid molecules seem to suggest possible sterol lipids for the molecules observed above *m/z* ^*–*^ 300, sterol lipids are rare for bacteria, yet not impossible (Zhai et al., 2024). Another possibility of the molecules observed above *m/z* ^*–*^ 300 is phospholipids. The molecule of *m/z* ^*–*^ 339.19 C_15_H_32_O_6_P^−^could likely be dodecylphosphoglycerol (DPG), a bacterial produced surfactant (Goncharuk et al., 2011; Li et al., 1997; Ryan et al., 1996). The observation of these phospholipids indicates that bacterial cell structural components and enzymatic activity in turn could degrade complex organic material into nutrients. The DPG can be absorbed and used for cellular functions within the cell membrane (Ramin and Allison, 2019). Unambiguous identification would need other corroborating evidence and possibly tandem MS to ensure positive assignment.

ToF-SIMS spectral results in Figure 2 show that the lipid production is dominant for the TSB media, whereas both the LB and the HOD media seem to fall behind. When the bacteria enter the stationary phases at 3 days, similar peaks are observed for the TSB and LB grown biofilms. This finding indicates that the bacteria begin to incorporate more of the organic matter and convert them into biofilms. The complex organic carbons provided by TSB and LB allow the bacteria to grow under less stressful and competitive environments. However, the HOD media spectrum at 3 days in Figure 3h, displays very high counts of palmitic acid, *m/z* ^*–*^ 255 C_16_H_31_O_2_^−^, and stearic acid, *m/z* ^*–*^ 283 C_18_H_35_O_2_^−^. Increases of palmitic and stearic acid are likely due to stress from low nutrient availability in the HOD media. However, the production of these molecules are also crucial in enhancing phosphorous solubilization (Benmrid et al., 2024). Phosphorous is an important molecule in nucleic acid production and cell division; and it is an essential macronutrient similar to nitrogen (Pan and Cai, 2023). Supplementary Figure S7 provides additional images of fatty acids.

Bacteria enter the death phase at the 7–days period, as indicated by the growth curve (Supplementary Figures S3, S4). While the molecular counts of lipids and fatty acids for the TSB and LB media are similar to the 3–days growth, the HOD growth biofilms show a decline in palmitic and stearic acids. However, the ion intensity of pentadecanoic acid *m/z* ^*–*^ 241 C_15_H_29_O_2_^−^ increases. The occurrence of pentadecanoic acid was linked to plant growth regulation, antimicrobial and antifungal properties, and production of fengycin (Al-Askar et al., 2024; Wang et al., 2024; Yin et al., 2024). The production of fengycin is an defense response, typically targeted against fungal infection (Dong et al., 2022). It is plausible the accumulation of pentadecanoic acid could be an indication of this defense response in relation to cellular stress or death leading to the observation of increased of pentadecanoic acid signal (Amarante-Mendes et al., 2018; Sur et al., 2018). Table 1 shows the proposed identifications of biological molecules at the 3-day period determined for each bacterial culture. Supplementary Table S5 shows biological molecule identifications for each time point in three media. Supplementary Table S6 shows the signal to noise ratio (SNR) values for each of the identified biological molecules discussed in the paper. SNR values above three are considered analytically different from background noise and can be considered real peaks (Alkemade et al., 1978; Shen et al., 2024). The peaks at *m/z* ^*–*^ 260.85 and *m/z* ^*–*^ 276.83 closely correspond to Na_3_S_2_O_8_^−^ and Na_3_S_3_O_7_^−^, which are possible sodium sulfate cluster ions. Bacterial cells in the presence of sodium sulfate were shown to cease cellular growth and eventually lead to cellular death (Nguyen and Kumar, 2022). While cellular production of sodium sulfate or sodium sulfate related products is not direct, indirect methods involving the metabolism of natural occurring sulfur containing molecules, such as amino acids, can release sulfate. If sodium is present, sodium sulfate can form as a result.

**TABLE 1:** ToF-SIMS possible peak identifications of biological material during 3-day growth in the mass range of *m/z* ^*–*^ 200–350.

*m/z*_−theo_	*m/z*_−obs_	ΔM, ppm	Species	Fragment	Assignment	HOD	LB	TSB
209.02442	209.027946	16.88248	$C_{13}H_{5}O_{3}^{-}$	[M-H]	Phenalene trione			x
211.04007	211.042469	11.38014	$C_{13}H_{7}O_{3}^{-}$	[M-H]	Hydroxyanthone		x	x
223.02166	223.033711	54.04743	$C_{6}H_{11}N_{2}O_{3}S_{2}^{-}$	[M-H]	Cysteylcysteine			x
227.20165	227.207733	26.759387	$C_{14}H_{27}O_{2}^{-}$	[M-H]	Myristic acid	x	x	x
241.2173	241.219864	10.6141	$C_{15}H_{29}O_{2}^{-}$	[M-H]	Pentadecanoic acid	x	x	x
255.23295	255.23487	7.508138	$C_{16}H_{31}O_{2}^{-}$	[M-H]	Palmitic acid	x	x	x
283.26425	283.265459	4.253915	$C_{18}H_{35}O_{2}^{-}$	[M-H]	Stearic acid	x	x	x
297.14962	297.159322	32.656963	$C_{19}H_{21}O_{3}^{-}$	[M-3H]	Sterol lipid		x	x
298.15744	298.161073	12.17538	$C_{19}H_{22}O_{3}^{-}$	[M-2H]	Sterol lipid			x
311.16527	311.176722	36.808633	$C_{20}H_{23}O_{3}^{-}$	[M-H]	Rubifolide		x	x
325.18092	325.192396	35.297392	$C_{21}H_{25}O_{3}^{-}$	[M-H]	Sterol lipid		x	x
339.19657	339.207971	33.6176	$C_{22}H_{27}O_{3}^{-}$	[M-H]	Sterol lipid		x	x

*m/z* ^−^. represents the mass-to-charge ratio for negatively charged.

m*/z* ^−^_theo._ represents the theoretical mass to charge ratio.

m*/z* ^−^_obs._ represents the observed mass to charge ratio.

ΔM, ppm is the mass deviation recorded for the observed *m/z* ^−^ against the theoretical *m/z* ^−^ in parts per million (Yu et al., 2023).

### 3.2 ToF–SIMS 2D imaging shows fatty acid distribution for HOD growth biofilms

The biofilm grown using the HOD media showed the most drastic changes in the three selected time points. This region of the SIMS spectra shown in Figure 2 corresponds to the most abundant peaks associated with fatty acids, lipids, and other small molecules that are EPS components. The increases in ion intensity for pentadecanoic acid, palmitic acid, stearic acid, and other lipid molecules provide some insight into the microbial ability of the 300A strain when inoculated via a minimal medium. 2D imaging of ToF–SIMS provides a more in–depth evaluation of fatty acid distribution as the biofilm progresses through its growth curve. Figure 3 shows normalized ToF–SIMS 2D images of select fatty acids. Data were normalized to the total ion intensity. For ease of presentation, the maximal value was set to an arbitrary value of 0.02. The fatty acids are superimposed using red, green, and blue (RGB) colors. The fatty acids selected are *m/z* ^*–*^ 255.23 C_16_H_31_O_2_^−^ palmitic acid (red), *m/z* ^*–*^ 283.26 C_18_H_35_O_2_^−^ stearic acid (green), and *m/z* ^*–*^ 339.19 C_22_H_27_O_3_^−^ or C_15_H_32_O_6_P^−^lipid (blue). While the biological molecules identified here are suspected to be from the EPS it is worth noting that the bacteria themselves and the cellular plasma membrane can be the source of these molecules.

The TSB medium at 1-day growth show the largest disparity, with palmitic acid on one side and the lipid molecule comprising the other side in Figure 3a. The 2D image of LB medium during 1-day growth (Figure 3b) shows primarily palmitic acid distribution. In contrast, the HOD medium image (Figure 3c) shows stearic acid as the primary molecule. The stearic acid could likely represent the nutrient competition when using a low carbon source medium. The 3-day growth period for each media (Figures 3d–f) shows a similar trend to that of the 1-day results. The TSB images show primarily palmitic acid and lipids, with some stearic acid with increased intensity. The LB results have an increase in stearic acid hot spots, similar to TSB. However, findings of the HOD medium are completely overtaken by stearic acid at 3-day growth. This drastic increase in stearic acid is likely due to stress responses. Consequently, the biofilm needs to modulate and limit resource exchanges, conserve energy, and promote survivability (Dubois-Brissonnet et al., 2016). The 7-day culture fatty acid and lipid distribution (Figures 3g–i) shares the same trend as the 3-day cultures for TSB and LB media. In contrast, the HOD medium results (Figure 3i) show an increase in palmitic acid and reduction in stearic acid. While the role of these fatty acids is not easy to be singled out, it is known that fatty acid production can have beneficial and harmful effects to bacterial cells. It is likely that higher productions of palmitic acid and stearic acid are direct results from high stress and low nutrient availability. The occurrence of fewer lipid molecules, as shown in HOD biofilms, could also be an indication of high stress and survivability mechanisms.

**FIGURE 4:**
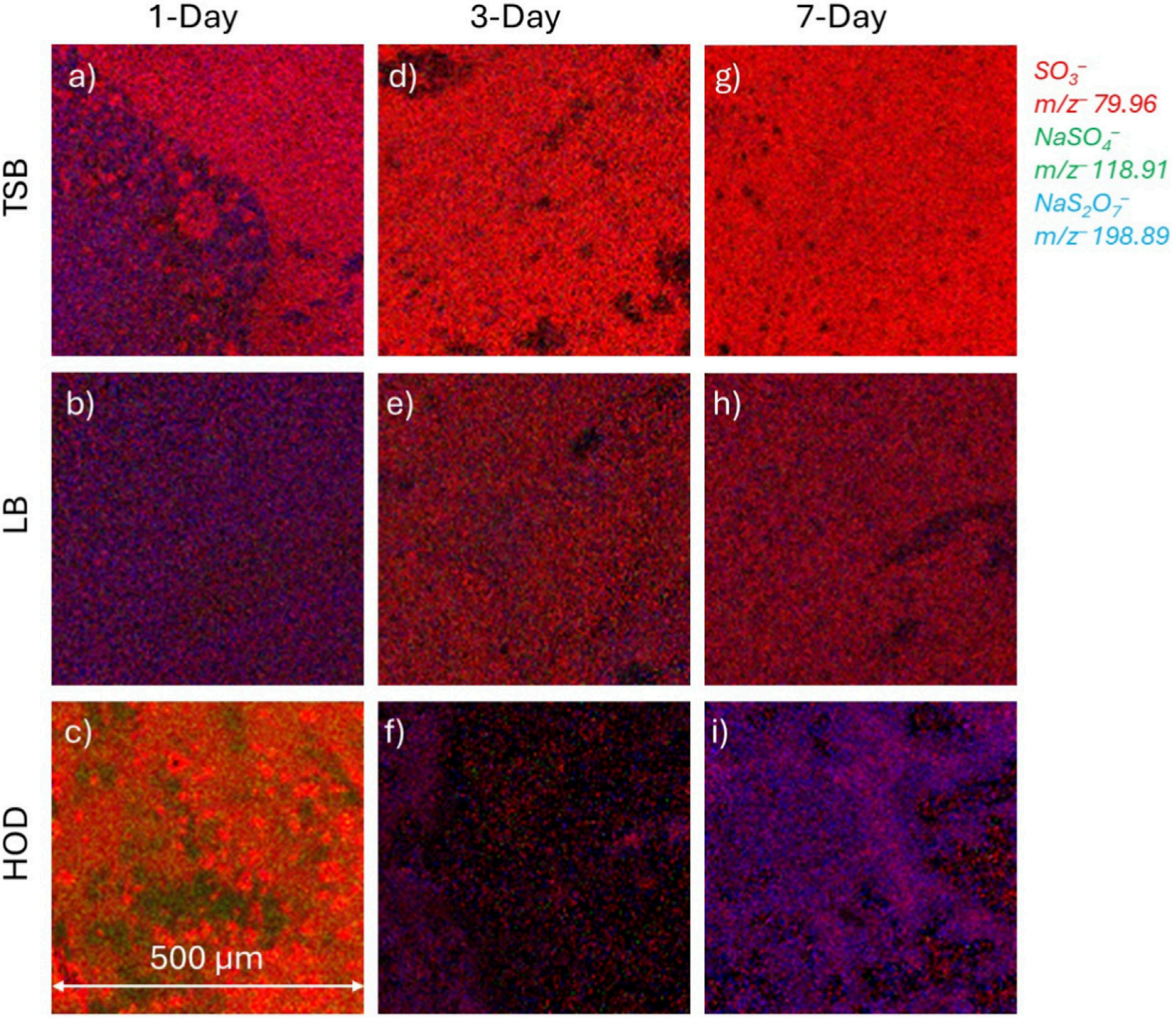
2D SIMS images of red, green, blue (RGB) showing overlays of sulfate molecules involved in cell deterioration, namely *m/z* ^−^ 79.96 SO_3_^−^ sulfite (red), *m/z* ^−^ 118.91 NaSO_4_^−^ sodium sulfate (green), and *m/z* ^−^ 198.89 NaS_2_O_7_^−^ sodium persulfate (blue). The formation of the sodium persulfate is likely to lead to the formation of death and stress observed in the cells. Images are normalized to total ion values. **(a,d,g)** correspond to TSB media growth during 1, 3, and 7–day periods. **(b,e,h)** correspond to LB media growth during 1, 3, and 7–day periods. **(c,f,i)** correspond to HOD media growth during 1, 3, and 7–day periods.

Sulfate reduced products and salts, such as *m/z* ^*–*^ 79.96 SO_3_^−^ sulfite, *m/z* ^*–*^ 118.91 NaSO_4_^−^ sodium sulfate, and *m/z* ^*–*^ 198.89 NaS_2_O_7_^−^ sodium persulfate among others, are also observed in 2D images of the HOD grown biofilms (Figure 4). Supplementary Figure S8 shows ToF-SIMS 2D imaging of individual species identified in Figure 4. The existence of these sodium sulfate and sodium persulfates can have varying influence on bacterial response. For instance, they can exhibit bacteriostatic effects, thus limiting the EPS production and increasing the cellular stress response (Balaberda and Ulrich, 2021; Irwin et al., 2017). SIMS 2D imaging can be used to visualize the biofilm’s stress response as shown in our results. Using the HOD medium, peaks, such as *m/z* ^*–*^ 118.91 NaSO_4_^−^ sodium sulfate and *m/z* ^*–*^ 198.89 NaS_2_O_7_^−^ sodium persulfate (Figures 4c,f,i) were found to be present in locations lacking stearic acid (see Figures 3C,F,I). In contrast, the TSB and LB media had little, or no sodium sulfate peaks in the spectral results. However, some sodium persulfate at each time point, 1-day, 3-day, and 7-day, was observed to overlap with the palmitic acid distributions (see Figure 3).

When comparing Figures 3a, 4a, the palmitic acid shown in red for Figure 3a corresponds to the same signal locations for sodium persulfate shown in blue in Figure 4a. In contrast, the lipid molecule of Figure 3a overlaps with the sulfite in Figure 4a. Figure 3a does not have much stearic acid in the analyzed region. This can be a representation of homeostasis. It is suspected that accumulation of palmitic acid may correspond to cellular stress which is why there is overlap of palmitic acid and sodium persulfate. Comparison of Figures 3c, 4c shows areas with high amounts of stearic acid in green, corresponding to areas with sulfite, similar to the previous comparison for the TSB medium. The areas shown in Figure 4c where NaSO_4_^−^ is identified could be from the media. Supplementary Figures S9, S10 show peak identifications of salts originating within the media. As shown in Figure 3d, stearic acid is located in pockets. The lipid molecule presented may indicate a healthier biofilm as sodium sulfate or persulfate are not observed in Figure 4d. However, as to the HOD medium grown biofilm at 3-Days, Figure 3f shows significant amounts of stearic acid, indicating that the biofilm is under extreme stress. Figure 4f shows more sodium persulfate in areas of stearic acid. This could be an indicator of the transition from the stationary phase towards the death phase. In Figures 3i, The occurrence of palmitic acid overlaps with sulfite and sodium persulfate in Figure 4i. The existence of stearic acid can be found in pockets but does not overlap with signal from sodium sulfate or sodium persulfate.

The existence of excess palmitic acid can be linked to lipid accumulation and induce oxidative stress and cellular death (Chen et al., 2024; Park et al., 2011; Uchiyama et al., 2025). This could be the reason why the sulfate and fatty acid compounds coexist in the biofilm. While not common, some species within the *Paenibacillus* genus are sulfate reducing bacteria (SRB), implying that these bacteria can metabolize sulfate and convert it to energy using the sulfate fermentation process (Li et al., 2017). The presence of sulfate and sulfate reduced ions, such as molecular sulfur, sulfur monoxide, sulfur dioxide and sulfite, observed in *Paenibacillus* sp. 300A may suggest that this bacterium belong to SRB. However, further studies are warranted to confirm this proposition as possible influence via sputtering could be inducing the reduction that is observed. SIMS spectral identification of sulfates is shown in Supplementary Figure S11.

### 3.3 Biofilm cultures show signs of stress in HOD minimal medium in 2D SIMS imaging

**FIGURE 5:**
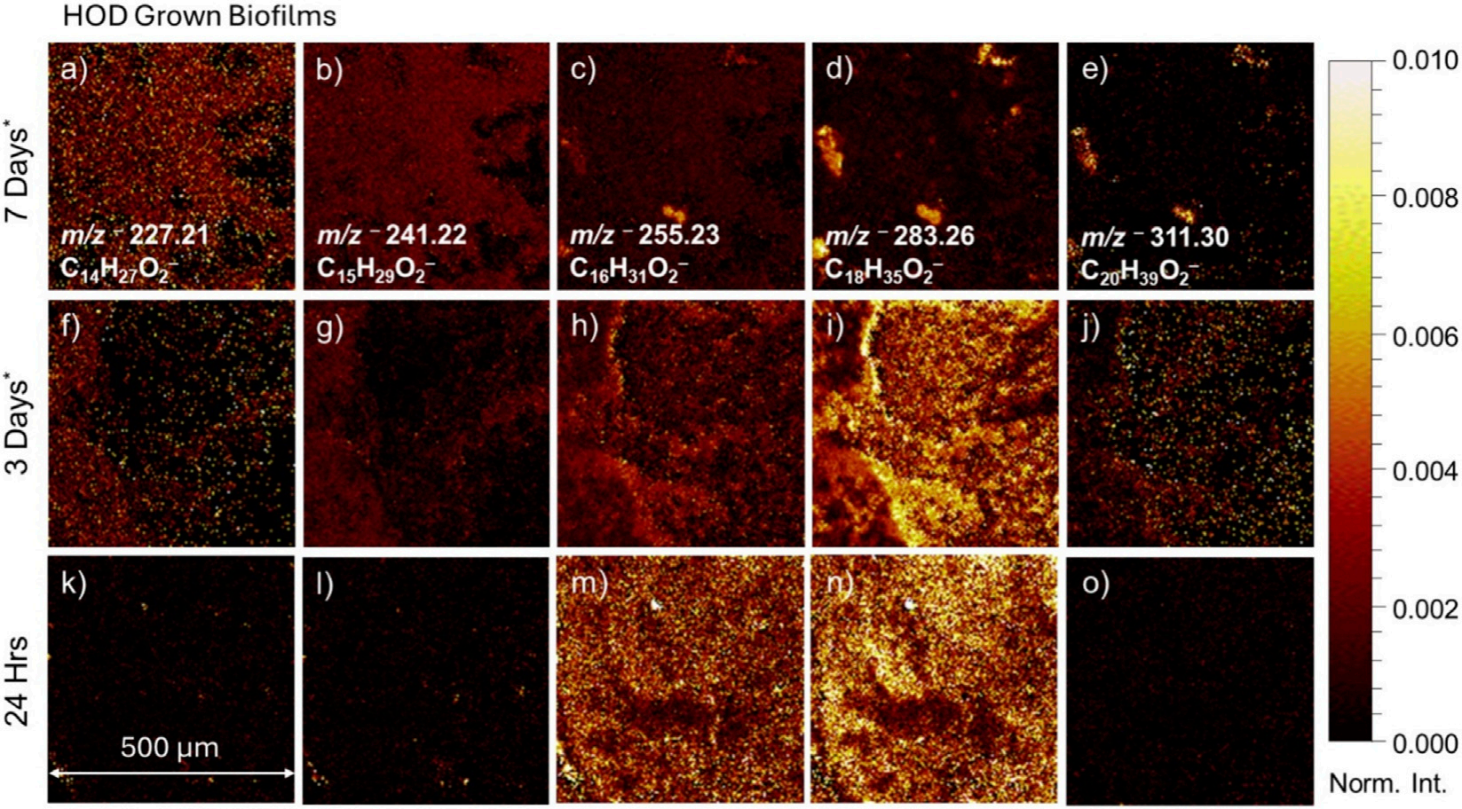
ToF–SIMS 2D images showing fatty acids *m/z* ^–^ 227.21 C_14_H_27_O_2_^–^ myristic acid (C14:0), *m/z* ^–^ 241.22 C_15_H_29_O_2_– pentadecanoic acid (C15:0), *m/z* ^–^ 255.23 C_16_H_31_O_2_^–^ palmitic acid (C16:0), *m/z* ^–^ 283.26 C_18_H_35_O_2_^–^ stearic acid (C18:0), and *m/z* ^–^ 311.30 C_20_H_39_O_2_^–^ arachidic acid (C20:0) from 1–day, 3–days, and 7–days, respectively for the HOD grown biofilms. Images are normalized to total ion values; panels d, e, g, h, j, and k are normalized to 0.05. **(a–e)** correspond to 7–day grown biofilms in HOD showing myristic acid, pentadecanoic acid, palmitic acid, stearic acid, arachidic acid. **(f–j)** correspond to 3–day grown biofilms in HOD showing myristic acid, pentadecanoic acid, palmitic acid, stearic acid, arachidic acid. **(k–o)** correspond to 24 hr (1–day) grown biofilms in HOD showing myristic acid, pentadecanoic acid, palmitic acid, stearic acid, arachidic acid.

The spectral and imaging results from the HOD-grown biofilms provide insight into the molecules when the bacterial cells are under stress due to lack of nutrients. The 2D distribution of other fatty acids observed in the HOD grown biofilms in SIMS spectral and 2D image analyses, such as *m/z* ^*–*^ 227.21 C_14_H_27_O_2_^−^, *m/z* ^*–*^ 241.22 C_15_H_29_O_2_^−^, *m/z* ^*–*^ 255.23 C_16_H_31_O_2_^−^, *m/z* ^*–*^ 283.26 C_18_H_35_O_2_^−^, *m/z* ^*–*^ 311.30 C_20_H_39_O_2_^−^, are shown in Figure 5. The 2D SIMS images, shown in Figure 5, are normalized to total ion intensity indicating a value of 0.010. However, due to the high peak intensity of some molecules, panels, d, e, g, h, j, and k are normalized to 0.050 to allow for easier viewing and comparison. During the 1-day growth evaluation, the biofilms show with limited detection of fatty acids besides *m/z* ^*–*^ 255.23 C_16_H_31_O_2_^−^ and *m/z* ^*–*^ 283.26 C_18_H_35_O_2_^−^ as indicated by Figures 5k–o. Once the biofilm entered the 3-day period (Figures 5f–j), significant biomass formation and clustering of fatty acid counts are observed spatially. The fatty acid peaks *m/z* ^*–*^ 227.21 C_14_H_27_O_2_^−^ and *m/z* ^*–*^ 311.30 C_20_H_39_O_2_^−^ seem to congregate close together, while the other fatty acid signals like *m/z* ^*–*^ 241.22 C_15_H_29_O_2_^−^, *m/z* ^*–*^ 255.23 C_16_H_31_O_2_^−^, and *m/z* ^*–*^ 283.26 C_18_H_35_O_2_^−^ seem to have more equal distribution across the surface. Once the bacteria entered the death phase at 7-day (Figures 5a–e), fatty acid distributions seem localized for *m/z* ^*–*^ 283.26 C_18_H_35_O_2_^−^ and *m/z* ^*–*^ 311.30 C_20_H_39_O_2_^−^. These phenomena are likely attributed to biofilm flocculations, which indicates that biofilms experience the most stress due to the low availability of nutrients while maintaining survivability (de Carvalho and Caramujo, 2018).

The ToF–SIMS spectra and 2D images reported throughout (e.g., Figure 2) show semi-quantitative findings that, over time in an anerobic environment, the biofilm grown in the HOD media was suitable to study and image characteristic fatty acids when compared to the traditional growth medias, TSB and LB. Additionally, the fatty acids observed in the HOD media seem to indicate that the bacteria are experiencing a level of stress not seen in the TSB or LB media. Other small molecular 2D images of as nitric oxide (*m/z* ^*–*^ 29.99 NO^−^), carbon dioxide (*m/z* ^*–*^ 43.99 CO_2_^−^), and chlorine (*m/z* ^*–*^ 34.97 Cl^–^) are shown in the Supplementary Figures S12, S13. These molecules can come from larger biological molecules, such as amino acids, fatty acids, and lipids. While they might also correspond to gaseous molecules, it is very difficult to differentiate the sources. Our analysis and peak identification is largely based on understanding of the biological system. It is also worth mentioning that shadowing effects often associated with molecular imaging are not believed to be evident within the presented 2D SIMS images. Shadowing and charging effects often make molecular imaging analysis challenging. Reduction of shadowing by careful sample preparation is desired. The HOD medium has been previously used to promote hydrogen (H_2_) driven reduction of nitrate for hydrogenotrophic growth (Lee et al., 2015). HOD promotes autotrophic growth using H_2_ during anoxic and denitrifying conditions. It is anticipated that HOD promotes lithoautotrophic growth. Autotrophic growth refers to the bacterial growth of an organism that produces its own energy or food source; whereas lithoautotrophic growth refers to organisms that derive energy from reduced mineral compounds (Natarajan and Prakasan, 2013; Stevens, 1997). Hydrogenotrophic growth uses H_2_ gas, and it is a subset of lithotrophic growth. This particular medium was recently used in the study of microbial influenced corrosion of glass material using *Paenibacillus polymyxa SCE2*. It was postulated that the HOD media might promote the corrosion of material so that the bacteria could undergo lithoautotrophic growth given the limited nutrient availability. It was demonstrated that glass could be used as a mineral source to sustain bacterial growth for up to 3 months, as supported by the detection of microbial influenced corrosion products (Parker et al., 2024).

## 4 Conclusion

Media selection and preparation were investigated using the *Paenibacillus* sp. strain model using ToF-SIMS spectral and 2D image analysis. Unlike other mass spectrometry imaging techniques, such as MALDI and DESI, SIMS spectroscopy measurements acquired under static limits cause no significant changes to the surface or chemically alter the sample. Our results show that the use of the minimum media has significant impact on the biofilm components, especially fatty acids and lipids that are EPS components and possibly bacterial plasma membrane. Without supplemental contributions to the media, the 300A biofilm enters the death phase after 95 h according to the measured growth curves. SIMS observation show that sodium sulfate salts are present during the death phase, which has been previously linked to biostatic conditions and cellular death. Increases in fatty acids, such as palmitic and stearic acid, may be attributed to cellular stress response and increase in specific regions of the biofilm, as evidenced in ToF–SIMS spectral analysis and 2D imaging results. Biofilm growth is supported by detection of EPS component peaks among the three media for the 3-day samples. SIMS spectral analysis and comparisons show significant differences for biofilms sampled at different time stamps relating to the biofilm phases.

Compared to rich media like TSB and LB, the HOD minimal medium’s primary function is to curtail the bacterial growth and limit the amount of initially available nutrient sources within the media. Our investigations show that the HOD medium allows for a realistic visualization of fatty acid and lipid production, simulating conditions bacteria are likely to encounter under limit resource availability. The use of HOD media to simulate bacterial growth is useful and important for understanding the biological molecules produced in biofilm colonization. Our SIMS spectral and imaging results suggest that HOD could serve as a good choice to study the effects of biofilms and their effects in resource lean conditions.

## Data Availability

The original contributions presented in the study are included in the article/Supplementary Material, further inquiries can be directed to the corresponding author.

## Licenses and permissions

This manuscript has been authored by UT-Battelle, LLC under Contract No. DE-AC05-00OR22725 with the U.S. Department of Energy. The United States Government retains and the publisher, by accepting the article for publication, acknowledges that the United States Government retains a non-exclusive, paid-up, irrevocable, world-wide license to publish or reproduce the published form of this manuscript, or allow others to do so, for United States Government purposes. The Department of Energy will provide public access to these results of federally sponsored research in accordance with the DOE Public Access Plan (http://energy.gov/downloads/doe-public-access-plan).
