# Supplementary material for: Molecular imaging uncovers growth media influence on biofilms’ EPS production
Source: Front Chem. 2026 Feb 11;13:1703055. doi: 10.3389/fchem.2025.1703055 (PMC12933274; doi:10.3389/fchem.2025.1703055)
Supplement: Supplementary file 1 [file DataSheet1.docx]

Supplementary Material

Molecular Imaging Uncovers Growth Media Influence on Biofilms’ EPS Production

Gabriel D. Parker^1,2^, Andrew Plymale^3^, Jacqueline Hager^3^, Luke Hanley^1^, and Xiao-Ying Yu^2*^

^1^Department of Chemistry, University of Illinois Chicago, Chicago, IL, USA

^2^Materials Science and Technology Division, Oak Ridge National Laboratory, Oak Ridge, TN, 37830, USA

^3^Earth Systems Science Division, Pacific Northwest National Laboratory, Richland, WA, 99352, USA

**Correspondence:**Xiao-Ying Yu, email: yuxiaoying@ornl.gov

Contents

[1 Supplementary Experimental Details S4](#_Toc213170342)

[1.1 Bacterial Identification Process: S4](#_Toc213170343)

[2 Supplementary Figures S5](#_Toc213170344)

[Figure S1: Prominent HOD medium control peaks and associated areas in 1-Day, 3-Day, and 7-Day biofilm cultures shows peak reduction. S5](#_Toc213170345)

[Figure S2: Detailed view showing different analytes, such as phosphite and fatty acids, mass deviation for 1-Day, 3-Day, and 7-Day biofilm growth using the HOD medium. S6](#_Toc213170346)

[Figure S3: *Paenibacillus sp. 300A* growth curve in HOD medium with glucose as carbon source under bulk aerobic conditions. S7](#_Toc213170347)

[Figure S4: Additional *Paenibacillus sp.* 300A biofilm growth curves in a) room temperature and b) 30 °C. S8](#_Toc213170348)

[Figure S5: Paenibacillus sp. 300A biofilms grown in TSB, LB and HOD medias for 24 hrs., 3 days, and 7 days. S9](#_Toc213170349)

[Figure S6: ToF-SIMS spectra of media controls within mass range *m/z^–^* 200-350 with normalized intensity values. S10](#_Toc213170350)

[Figure S7: ToF-SIMS 2D images showing different fatty acid (C16:0, C18:0, & C20:0) distribution across the substrate as grown via various media. S11](#_Toc213170351)

[Figure S8: ToF-SIMS 2D imaging showing different sulfate representations (SO_3_^–^, NaSO_4_^–^, & NaS_2_O_7_^–^) distributions across the substrate as grown via various media. S12](#_Toc213170352)

[Figure S9: Peak identification of sulfate salts, NaSO_4_^–^, MgSO_4_^–^, FeSO_4_^–^, and CuSO_4_^–^ originating within the HOD medium and compared against the biofilms grown at 1-Day, 3-Day, and 7-Day periods. S13](#_Toc213170353)

[Figure S10: Peak identifications for sodium sulfate molecules observed in 1-Day, 3-Day, and 7-Day biofilms. S14](#_Toc213170354)

[Figure S11: Sulfate and sulfate reduced products such as sulfite, sulfur dioxide, sulfur monoxide, and molecular sulfur observed in the spectra of biofilms cultures using each medium (TSB, LB, HOD) at time points 1-Day, 3-Days, and 7-Days. S15](#_Toc213170355)

[Figure S12: ToF-SIMS 2D imaging showing small molecule (NO^–^, CO_2_^–^, & Cl^–^) distributions across the substrate as grown via various media. S16](#_Toc213170356)

[Figure S13: RGB overlay of nitric oxide NO^–^(red), carbon dioxide CO_2_^–^(green), and chlorine Cl^–^(blue) with intensity values normalized to 0.2 total ion intensity. S17](#_Toc213170357)

[Figure S14: Normalized ToF-SIMS 2D images showing spatial distribution of sodium sulfate molecules observed within the spectra and increasing intensity at 7-Day growth correlating with cellular death. S18](#_Toc213170358)

[3 Supplementary Tables S19](#_Toc213170359)

[Table S1: Hydrogen oxidizing de-nitrifier bacterial growth media constituent concentrations. S19](#_Toc213170360)

[Table S2: Concentrations of Wolfe’s vitamins and minerals included in the hydrogen oxidizing de-nitrifier bacterial growth media. S20](#_Toc213170361)

[Table S3: Tryptic soy broth and LB broth media composition. S21](#_Toc213170362)

[Table S4: Peak Reduction for Media Related Prominent Peaks. S22](#_Toc213170363)

[Table S5: Peak identifications for 1-day, 3-day, and 7-day growth for HOD, LB, and TSB growth media in the mass range of *m/z^–^* 200 – 350. S23](#_Toc213170364)

[Table S6: Signal to noise ratio (SNR) values for peak identifications of 1-day, 3-day, and 7-day growth using HOD, LB, and TSB growth media in the mass range of *m/z^–^* 200 – 350. S25](#_Toc213170365)

[4 References S27](#_Toc213170366)

# Supplementary Experimental Details

## Bacterial Identification Process:

The identification procedure followed a multi-step process. The IONTOF peak searching and mass matching functions are used, but not the IONTOF database. The mass matching function calculates different combinations of periodic table elements to mass match a selected peak to provide a mass deviation and match score. Using the “peak search” function within *SurfaceSpectra*, with parameters of SNR 3.0, max background 0.8, and minimum counts 25, a peak list was generated giving the best mass matching formula for the given *m/z* value under each specific sample. Sample peak values within ± *m/z* 0.05 of media peak values were removed. This value of ± *m/z* 0.05 was chosen to compensate for slight variations between analysis of sample and media. The biofilm or planktonic sample had spectral features that overlapped with those of the media. If there were standout values above ± *m/z* 0.05, then they were treated as unique to the biofilm or planktonic cell sample. The goal was to maintain only biological peaks not relating to media or other inorganic artifacts. It should be noted that large peaks within the ToF-SIMS spectra that correspond to media related peaks and other matrix effects are not identified or included within the peak tables. While mass matching is important in peak identification, it is not the only factor considered when assigning peaks. For example, *m/z^–^* 311.1741 is identified as C_20_H_23_O_3_^–^, but it can also be assigned as C_12_H_21_N_7_O_3_^–^, C_15_H_25_N_3_O_4_^–^, or C_13_H_21_N_5_O_4_^–^ if we take into consideration the isotopic ratios, the surrounding spectra, sample information, and the charge of the ion. While the mass values for C_12_H_21_N_7_O_3_^–^, C_15_H_25_N_3_O_4_^–^, and C_13_H_21_N_5_O_4_^–^ match closely with 311.1741, these molecules are peptides in their non-ionized form. On the other hand, C_20_H_23_O_3_^–^ is the anion of C_20_H_24_O_3_, which could be a hydroxybenzoate enzyme. When considering the charge of the molecule, there are now three other possibilities which the mass 311.1741 could be labelled. It could be C_13_H_28_O_6_P^–^, C_17_H_27_O_3_S^–^ or C_19_H_23_N_2_O_2_^–^. C_13_H_28_O_6_P^–^ is a glycerophospholipid and could be a possibility, C_17_H_27_O_3_S^–^ is a benzenoid benzenesulfonic acid which has a surfactant role, and C_19_H_23_N_2_O_2_^–^ is a cyclic dipeptide consisting of two arginine. The chemistry of the molecule and possible fragmentations of the are then considered. Based on mass deviation, molecule reporting, and molecular structure, C­_20_H_23_O_3_^–^ and C_13_H_28_O_6_P^–^ are the likely candidates. Further analysis via tandem mass spectrometry could identify the exact molecular composition of the peak at *m/z^–^* 311.1741. Major species identified within the spectra here are amino acids, fatty acids, lipids, and other relevant molecules. This finding is in agreement with previous results.(Ding et al., 2016;Zhang et al., 2020)

# Supplementary Figures


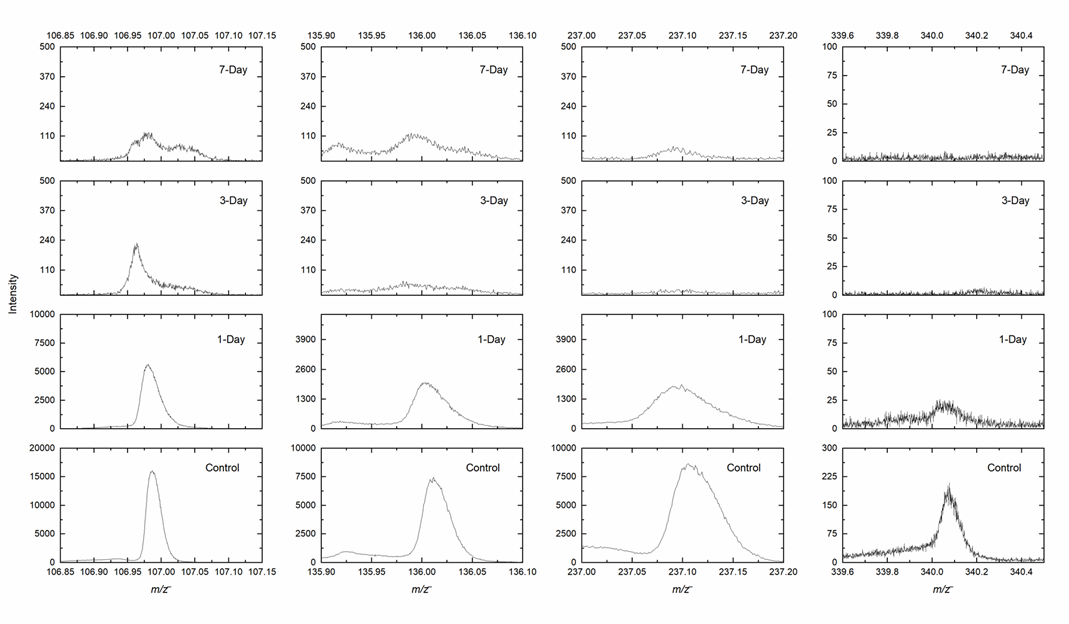


Figure S1: Prominent HOD medium control peaks and associated areas in 1-Day, 3-Day, and 7-Day biofilm cultures shows peak reduction.

**Figure S1** shows peak area reduction for the biofilm samples labeled as 1-Day, 3-Day, and 7-Day within the spectra comparing the control data. A main peak for control is HEPES at *m/z^–^* 237.11. The HEPES peak is reduced for the biofilm samples at the low end of 73.5% in the 1-Day biofilm sample and at the high end of 99.6% for the 3-Day biofilm samples. Peak reductions shows that the centrifugal spinning process of desalination/washing removes the media from the sample. Peak area reduction percentages can be found withing **Table S5**. It is worth noting that the 1-day growth does contain some medium peaks, but the percent reduction was approximately 50%.


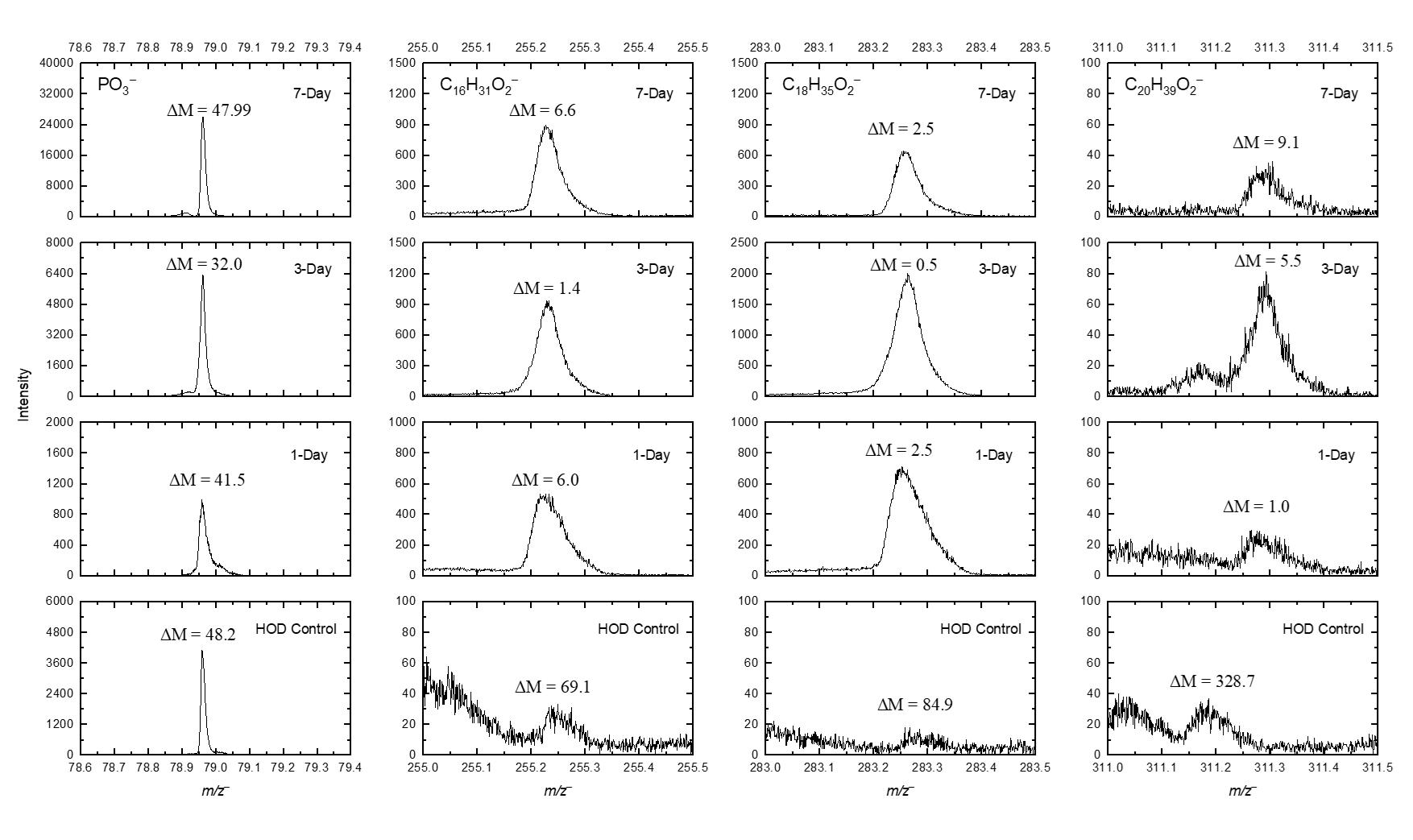


Figure S2: Detailed view showing different analytes, such as phosphite and fatty acids, mass deviation for 1-Day, 3-Day, and 7-Day biofilm growth using the HOD medium.

**Figure S2** depicts the calibration of the ToF-SIMS spectral data, showing that both the low mass values (e.g., PO_3_^–^) and high mass values have very good mass accuracy. It is important to know that fatty acids, such as C_16_H_31_O_2_^–^, C_18_H_35_O_2_^–^, and C_20_H_39_O_2_^–^, are native to the biofilm and they do not produce high intensity signal within the control sample. Thus, they can be used to provide the high mass deviation indicated in the graph. Phosphite, PO_3_^–^, is evident in both control and biofilm samples. The mass deviation is within acceptable range, ∆M <65ppm, for identification. **Figure S2** indicates that the calibration points chosen are suitable to obtain valid information. While not shown, the TSB and LB medium controls and biofilms have similar mass accuracy.


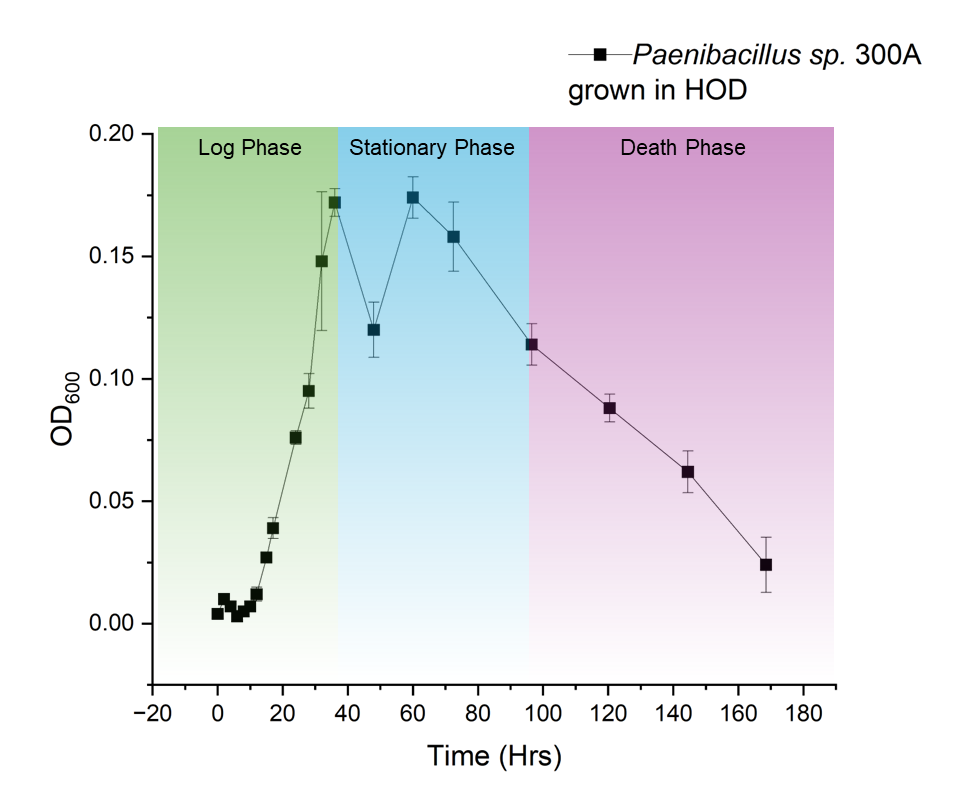


Figure S3: *Paenibacillus sp. 300A* growth curve in HOD medium with glucose as carbon source under bulk aerobic conditions.

**Figure S3** shows the growth curve of *Paenibacillus sp. 300A* with the exponential phase (green) beginning from zero hours to approximately 35 hours. The bacteria then enter the stationary phase (blue) this is captured from 35 to 95 hours. The stationary phase does show a slight dip in optical density before increasing. It is after 95 hours that the bacteria are expected to enter the death phase (purple) where cells die faster than being created. We observe the death phase as the OD_600_ begins to drop rapidly after 95 hours. From the beginning of the stationary phase at 35 hours to the beginning of the death phase at 95 hours there is a 33.7% percent decrease in optical density. A more detailed examination of growth would be needed to determine the exact moment the death phase begins and the stationary phase ends. However, the time periods 1-day, 3-days, and 7-days best represent our incubation process which correlate to the growth curve.


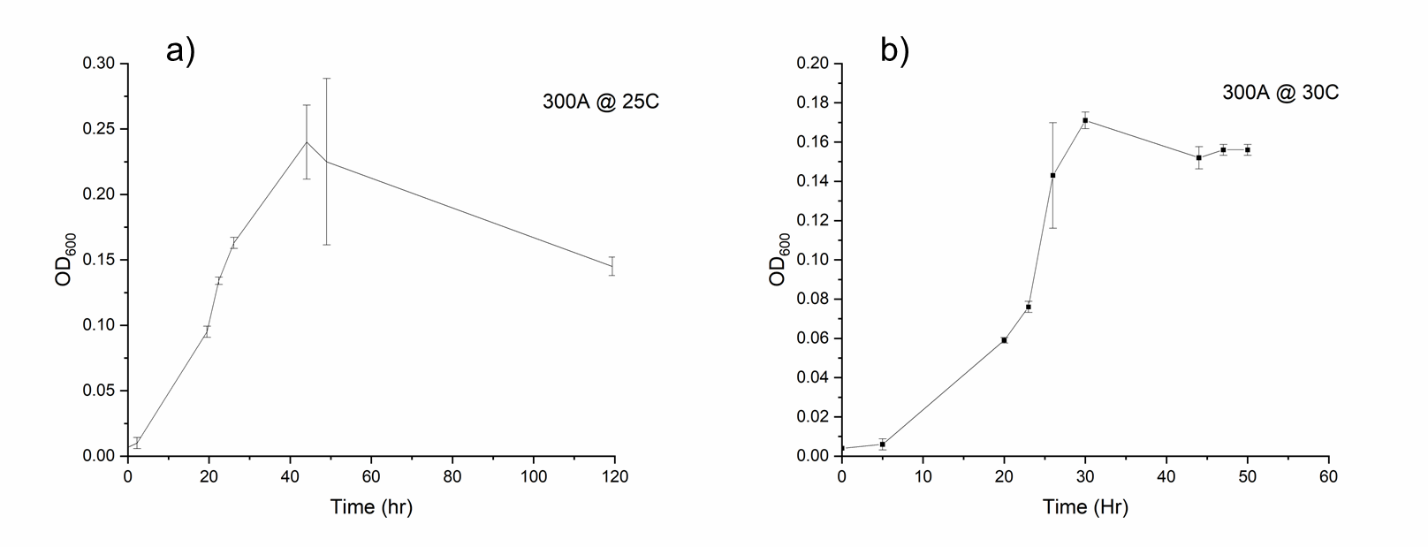


Figure S4: Additional *Paenibacillus sp.* 300A biofilm growth curves in a) room temperature and b) 30 °C.

**Figure S4** provides additional growth curves for the *Paenibacillus sp.* 300A bacterium. These growth curves show similar trends to those observed in **Figure S3.** In **Figure S4a**, the log phase stops around 42 hours. At 50 hours, there is a large standard deviation indicating that one replicate continued to increase while the other decreased, as these growth curves are performed in tandem with two replicates. To the extent of the period that the bacteria spent within the stationary phase, the data is unclear. However, it is noted that, at the final time point of 120 hours, the bacteria are in the death phase since the OD_600_ was reduced. In **Figure S4b**, the log phase stops at approximately 30 hours before entering the stationary phase which was only recorded until approximately 50 hours. The growth curve for **Figure S4b** was recorded at 30 °C, whereas the growth curves in **Figures S3** and **S4a** were obtained in room temperature, 25 °C. A higher temperature has a direct correlation with growth rate. We assume that, in room temperature the stationary phase extends beyond what is observed in **Figure S4b**, and likely past 72 hours.


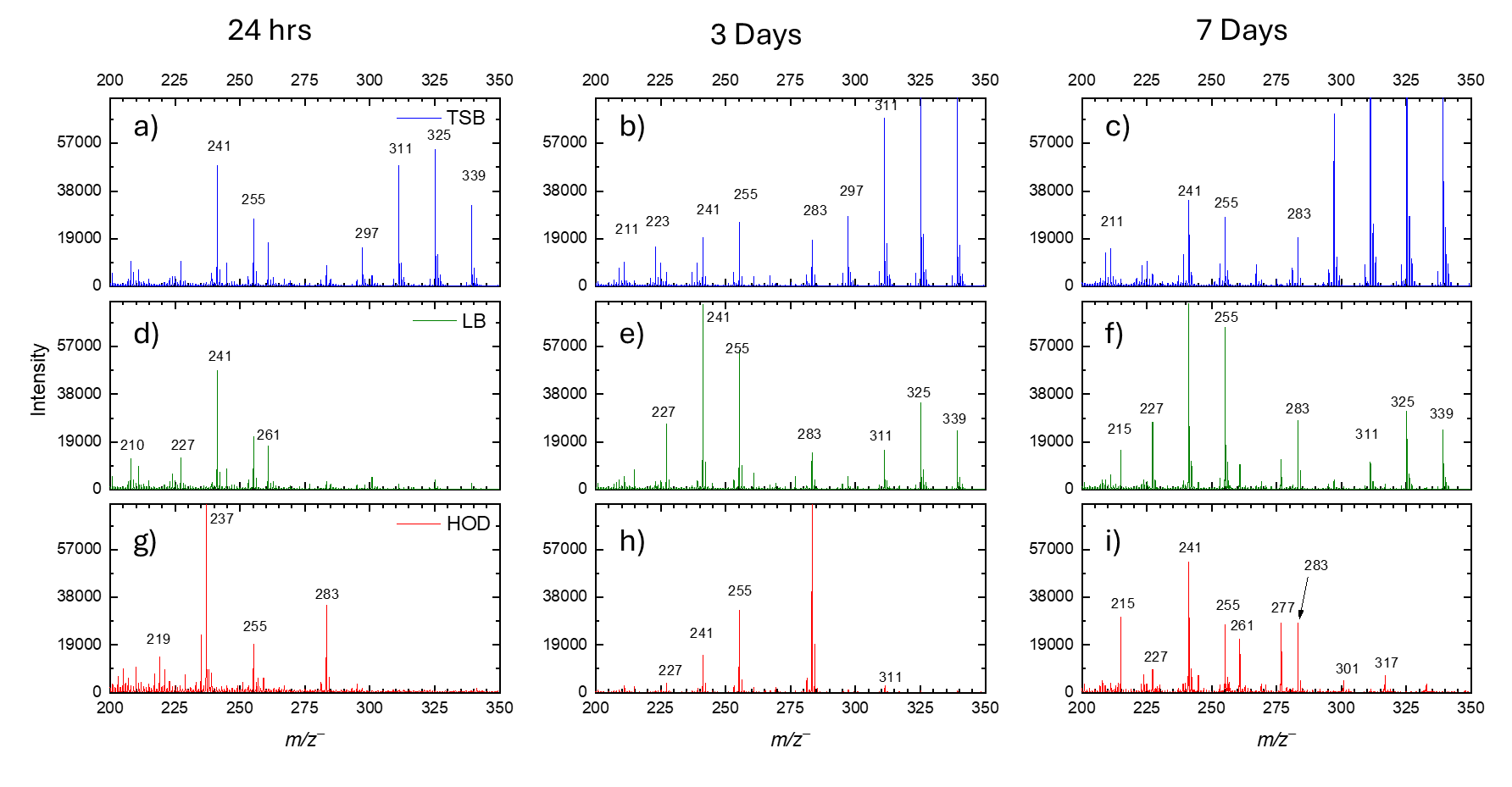


Figure S5: Paenibacillus sp. 300A biofilms grown in TSB, LB and HOD medias for 24 hrs., 3 days, and 7 days.

**Figure S5** supports the main text **Figure 4** by showing the true intensity values of the spectra collected. The values are normalized in **Figure 4** to show comparison across sample variance and spot to spot fluctuations. We notice that the HOD values of note are *m/z^–^* 227, 241, 255, and 283 most of which are fatty acids comprising the EPS. The LB media has similar values but shows more fatty acids and lipid molecules than the HOD. The same goes for TSB, which has the highest intensity values for many of the fatty acids and lipids. The main difference among the three media is the *m/z^–^* 283 signals for the HOD media. This is a prominent fatty acid, stearic acid, and has been shown to have links to cellular fatty acid incorporation during times of stress.(Baumgarten et al., 2012;Uruén et al., 2020;Atasoy et al., 2024). Since the HOD is described as a minimal medium, we hypothesize that the biofilms production of stearic acid is a defense mechanism; and its responses to unfavorable conditions by reducing lipid content for cellular regulation. It is anticipated that fatty acids would behave in this manner; and that stearic acid could be a marker for cellular stress response and an indicator of when cellular death may occur. **Figures S5g,h,i** show the trend of stearic acid in the HOD media. The relative abundance of steric acid increases from 1-Day to 3-Day growth and decreases significantly from 3-Day to 7-Day growth. As to the 7-Day growth biofilms, other markers, such as *m/z^–^* 261 and 267, arise and they are postulated to be fragments of sodium sulfate molecules.


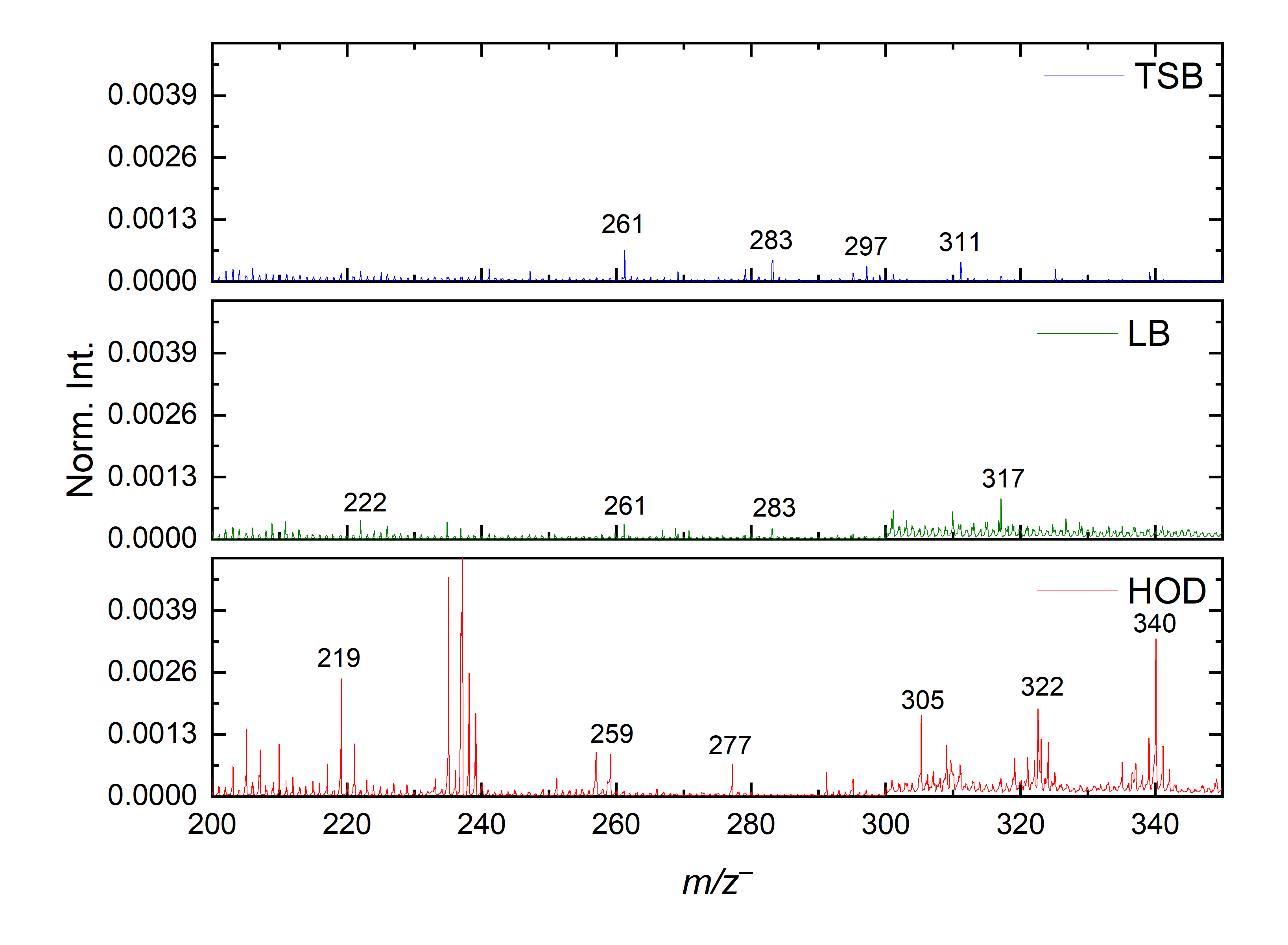


Figure S6: ToF-SIMS spectra of media controls within mass range *m/z^–^* 200-350 with normalized intensity values.

**Figure S6** shows normalized intensity ToF-SIMS spectra for the three media used in this experiment. The values are normalized to the values within the main text. While there are some overlaps with identifications within the spectra, namely, *m/z^–^ 219, 261, 283, 297, 311,* increases in ionization for values in the main text are expected to arise from the biofilm fatty acids and lipids and not the media. While the media may have some of these species within their digest, the peaks identified within the main text arise from the bacteria thus increasing the intensity of the observed values.


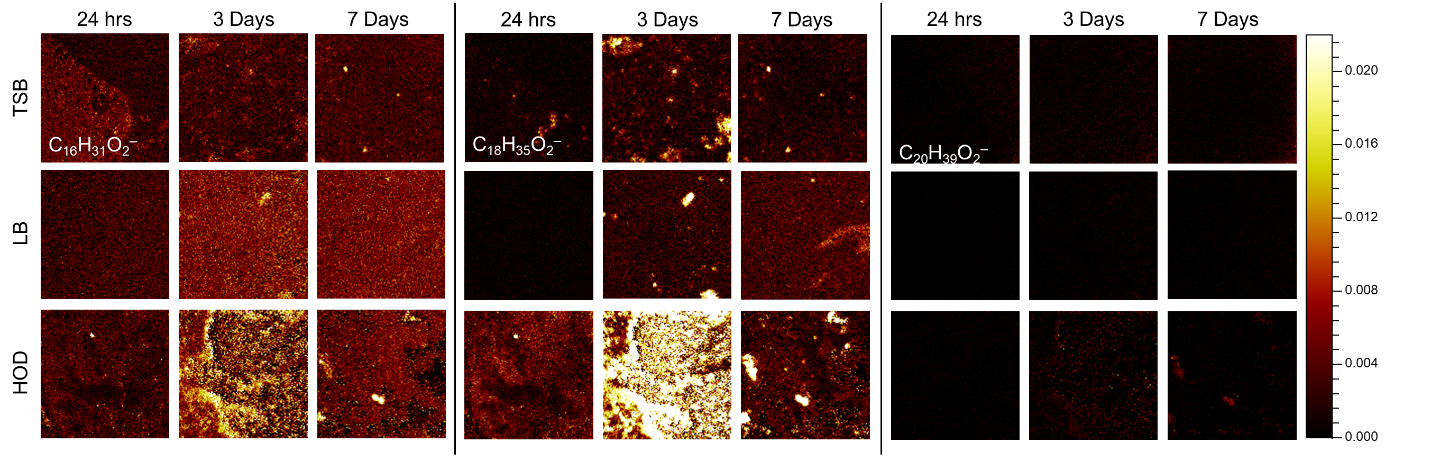


500 μm

Figure S7: ToF-SIMS 2D images showing different fatty acid (C16:0, C18:0, & C20:0) distribution across the substrate as grown via various media.

**Figure S7** shows three different fatty acids (*m/z^–^* 255.23 C_16_H_31_O_2_^–^, *m/z^–^* 283.26 C_18_H_35_O_2_^–^, *m/z^–^* 311.29 C_20_H_39_O_2_^–^) distribution over 1-day, 3-day, and 7-day periods. We notice that the higher weight fatty acid 311.29 C_20_H_39_O_2_^–^ does not register for the LB or the TSB and is very faint for the HOD growth media. It is expected that lipid molecules in this region are more prevalent than fatty acids. The fatty acids are observed to be in the highest intensity at the 3-day time period. This is consistent with the growth curve as the bacteria enters the stationary phase near the 3-day mark. **Figure S7** supports the main text **Figure 3**.


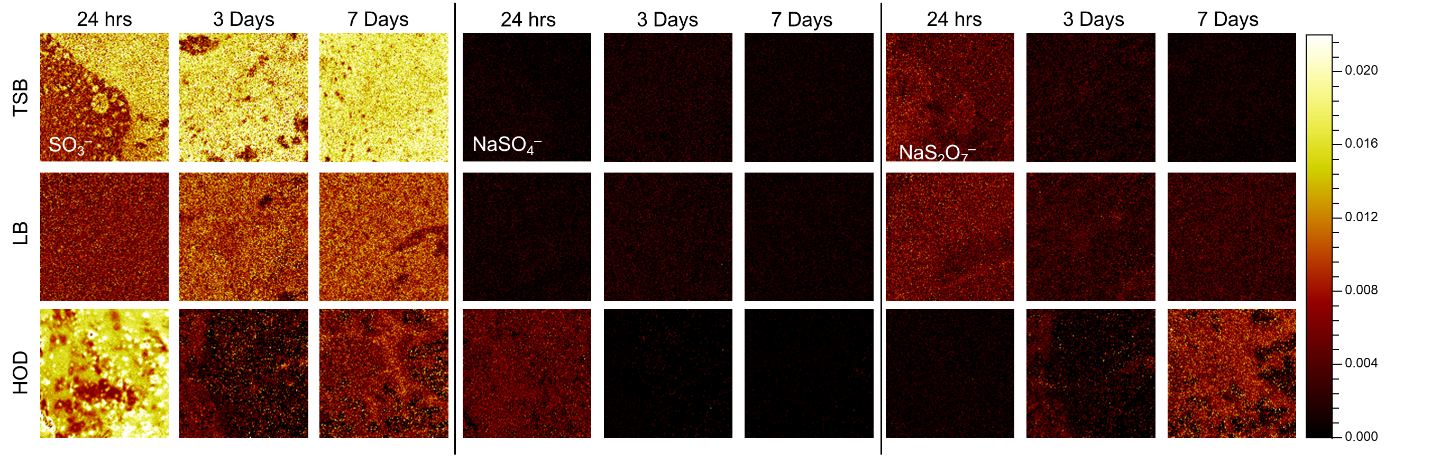


500 μm

Figure S8: ToF-SIMS 2D imaging showing different sulfate representations (SO_3_^–^, NaSO_4_^–^, & NaS_2_O_7_^–^) distributions across the substrate as grown via various media.

**Figure S8** shows the distribution of sulfate molecules observed in the biofilm. Sulfite (*m/z^–^* 79.96 SO_3_^–^), sodium sulfate (*m/z^–^* 118.93 NaSO_4_^–^) and sodium persulfate (*m/z^–^* 198.89 NaS_2_O_7_^–^) are believed to be biological and are observable during times of stress and cellular death. However, sulfite and sodium sulfate can also be a product of bacterial fermentation, the ATP process, or from the media components. The sodium persulfate does not originate in the media. Both sodium sulfate and sodium persulfate are associated with cellular death and polyaromatic hydrocarbon degradation, respectively.(Liao et al., 2018;Nguyen and Kumar, 2022). **Figure S8** supports **Figure 4** in the main text.


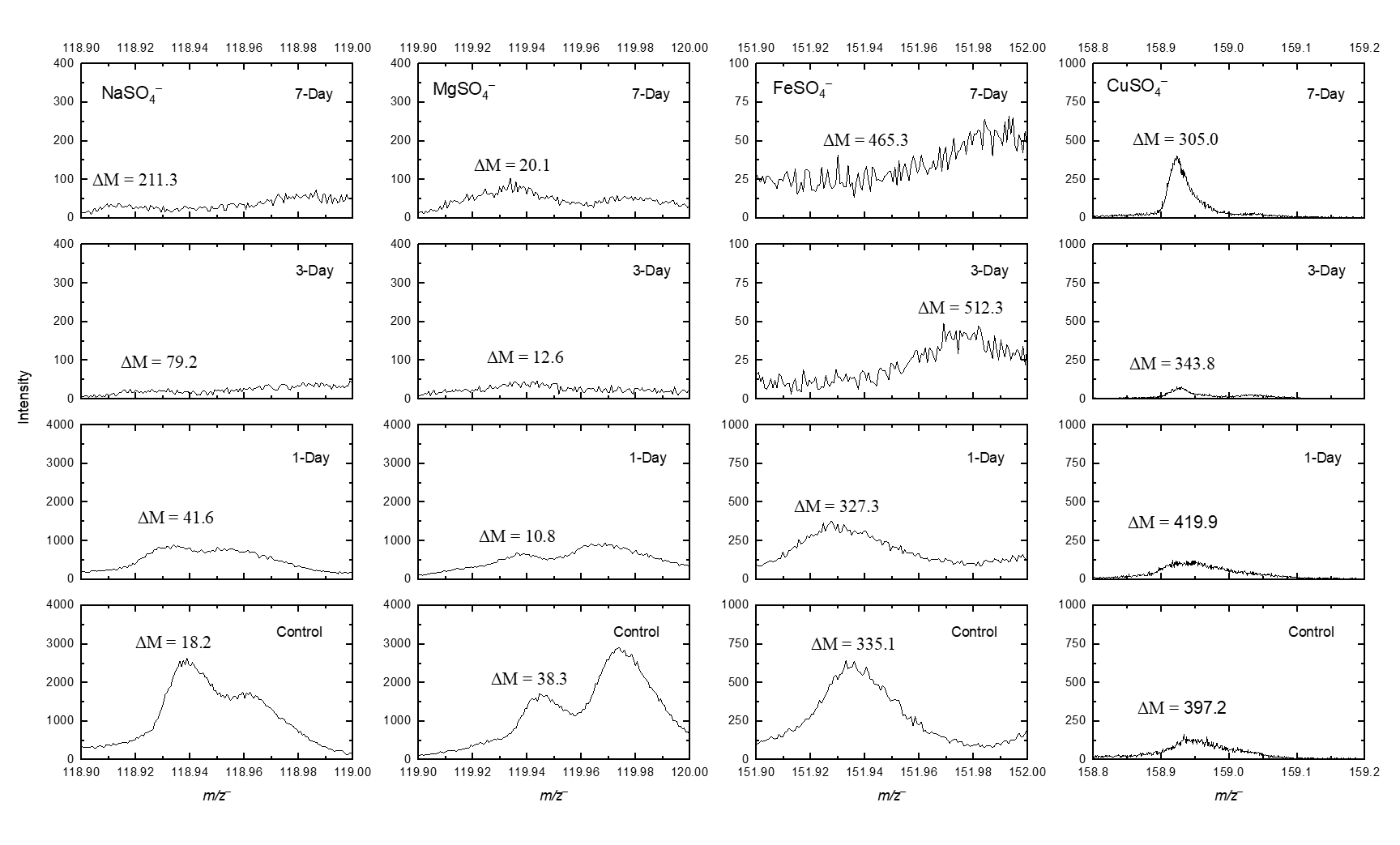


Figure S9: Peak identification of sulfate salts, NaSO_4_^–^, MgSO_4_^–^, FeSO_4_^–^, and CuSO_4_^–^ originating within the HOD medium and compared against the biofilms grown at 1-Day, 3-Day, and 7-Day periods.

**Figure S9** shows possible identification of salts originating from the HOD medium within the biofilms. While no sodium sulfate was listed in the HOD medium contents, there is a peak within the control that corresponds to NaSO_4_^–^ with a mass deviation of 18.2 ppm. It is worth noting that the presence of NaSO_4_^–^ weakens over the three time periods and the mass accuracy worsens. This could indicate that NaSO_4_^–^ is not within the biofilm but originates from the media. Similar results are obtained for MgSO_4_^–^, FeSO_4_^–^, and CuSO_4_^–^. Mass accuracy shows MgSO_4_^–^ within the media control and within the 1-Day grown biofilm; however, low ion counts make it difficult to determine if this identification can be conveyed for the 3-Day and 7-Day biofilms. As for FeSO_4_^–^, and CuSO_4_^–^, it is shown that the peaks identified within the areas do not correspond to the sulfates as the mass deviation is too high. Possible identifications typically need a mass deviation of 100 ppm or less, isotopic distribution or logical fragmentation, above 500 ion counts, and reasonable identification of the peak should be supported by literature or knowledge of the system.


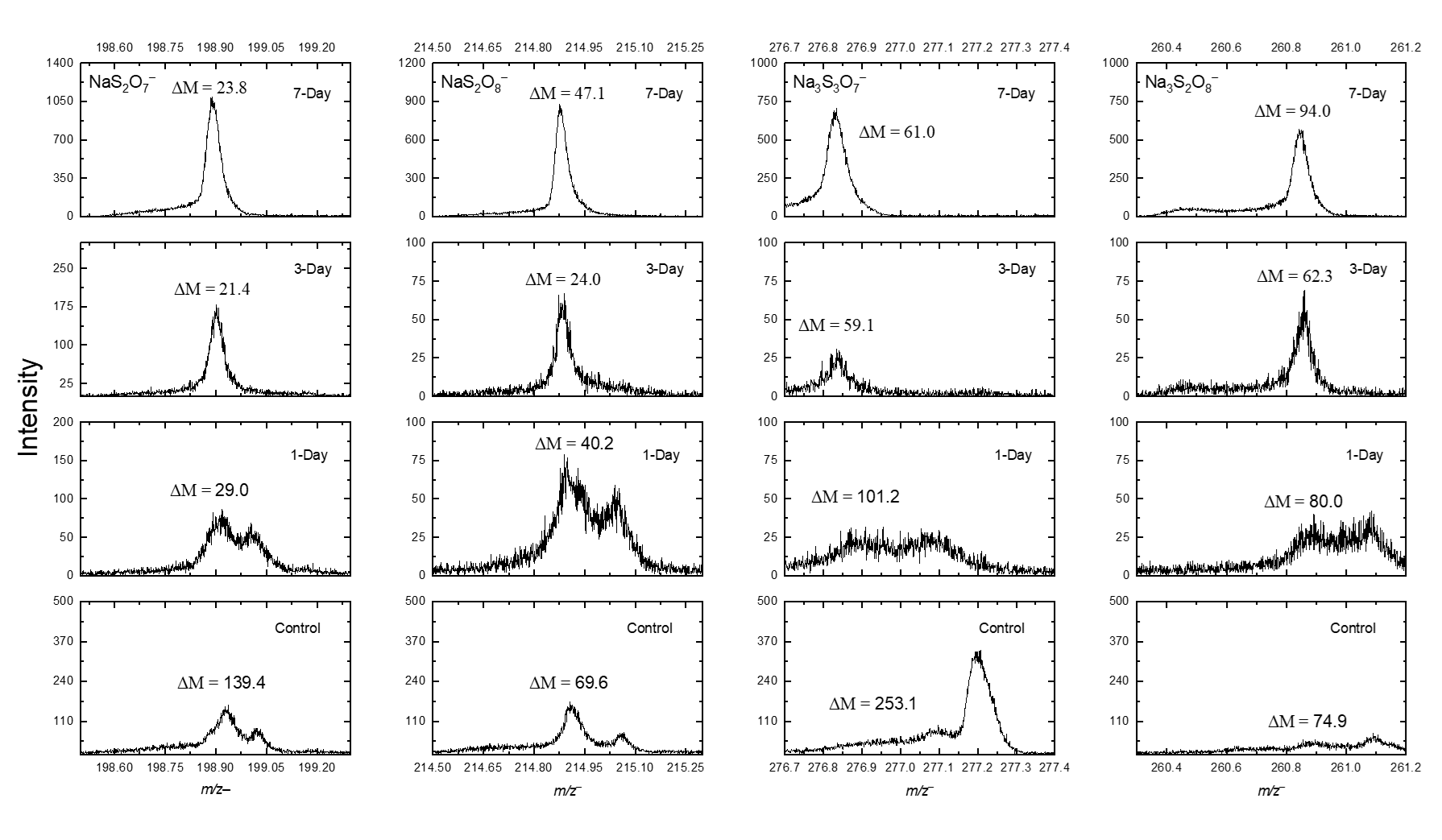


Figure S10: Peak identifications for sodium sulfate molecules observed in 1-Day, 3-Day, and 7-Day biofilms.

**Figure S10** identifies larger sodium sulfate molecules such as NaS_2_O_7_^–^, NaS_2_O_8_^–^, Na_3_S_3_O_7_^–^ and Na_3_S_2_O_8_^–^ of which are either not accurately identified within the control HOD medium or have high mass deviation in the control medium. As stated in the main text, these molecules show increase in intensity when measured on the 7-Day growth contributing to the reasoning linking sodium sulfate molecules as stressors and contributors to cellular death.

^
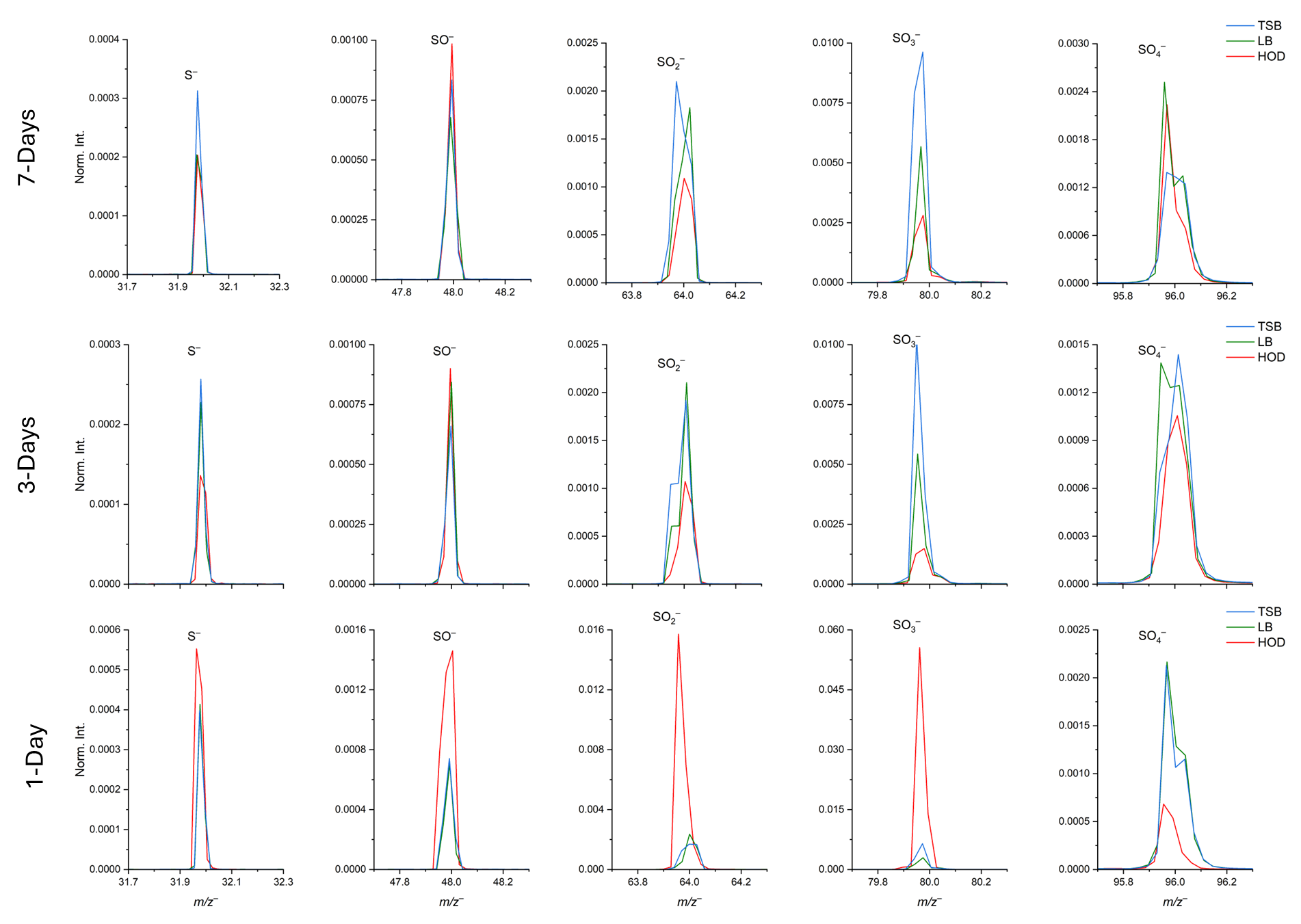
^

Figure S11: Sulfate and sulfate reduced products such as sulfite, sulfur dioxide, sulfur monoxide, and molecular sulfur observed in the spectra of biofilms cultures using each medium (TSB, LB, HOD) at time points 1-Day, 3-Days, and 7-Days.

**Figure S11** shows the ToF-SIMS spectral identifications of sulfate reduced products such as sulfite, sulfur dioxide, sulfur monoxide, and molecular sulfur. These identifications provide context for the observations of sodium sulfate and other sulfate products observed in the higher mass range. Note that sulfite (*m/z*^–^79.96 SO_3_^–^) is observed to have lager signal than sulfate (*m/z^–^*95.96 SO_4_^–^) for 1-day culture. The sulfite is higher than sulfate for 3-day and 7-day culture as well, but both have similar intensity. Identification of the reduced sulfate products could be indicative of sulfate reducing ability by the *Paenibacillus* bacterium; however, further analysis would be required to identify *Paenibacillus sp.* 300A as a sulfate reducing bacteria. It is worth noting that sputtering of the sample could also induce this effect, which was observed and has been taken into consideration.


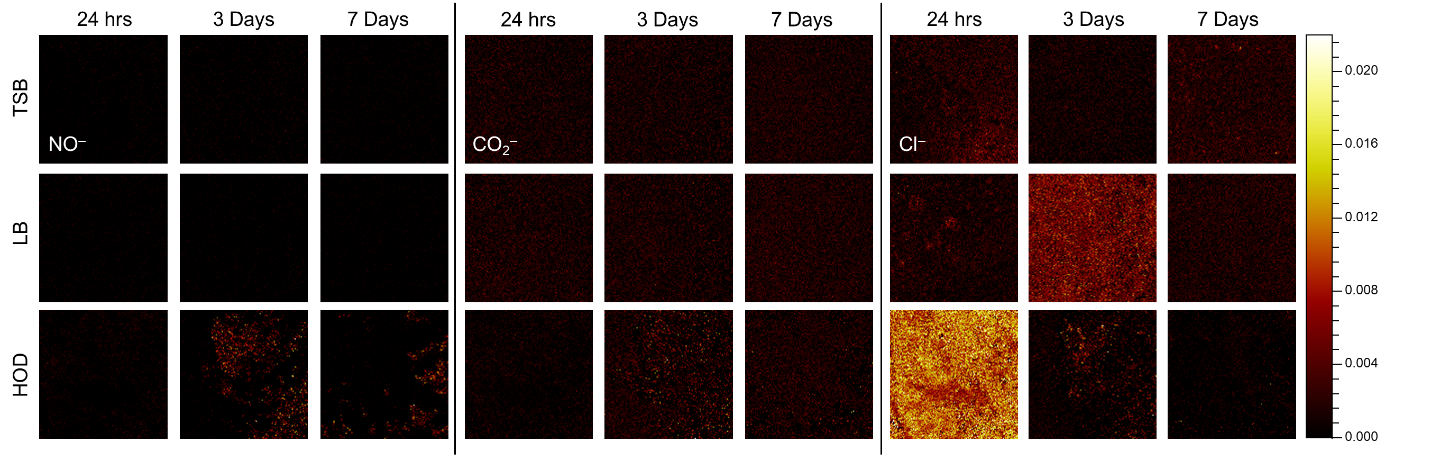


500 μm

Figure S12: ToF-SIMS 2D imaging showing small molecule (NO^–^, CO_2_^–^, & Cl^–^) distributions across the substrate as grown via various media.

**Figure S12** shows the distribution of small molecules typically found within biofilms, such as nitric oxide (*m/z^–^* 29.99 NO^–^), carbon dioxide (*m/z^–^* 43.99 CO_2_^–^), and chlorine (*m/z^–^* 34.97 Cl^–^). The scanning area is 500 μm by 500 μm.


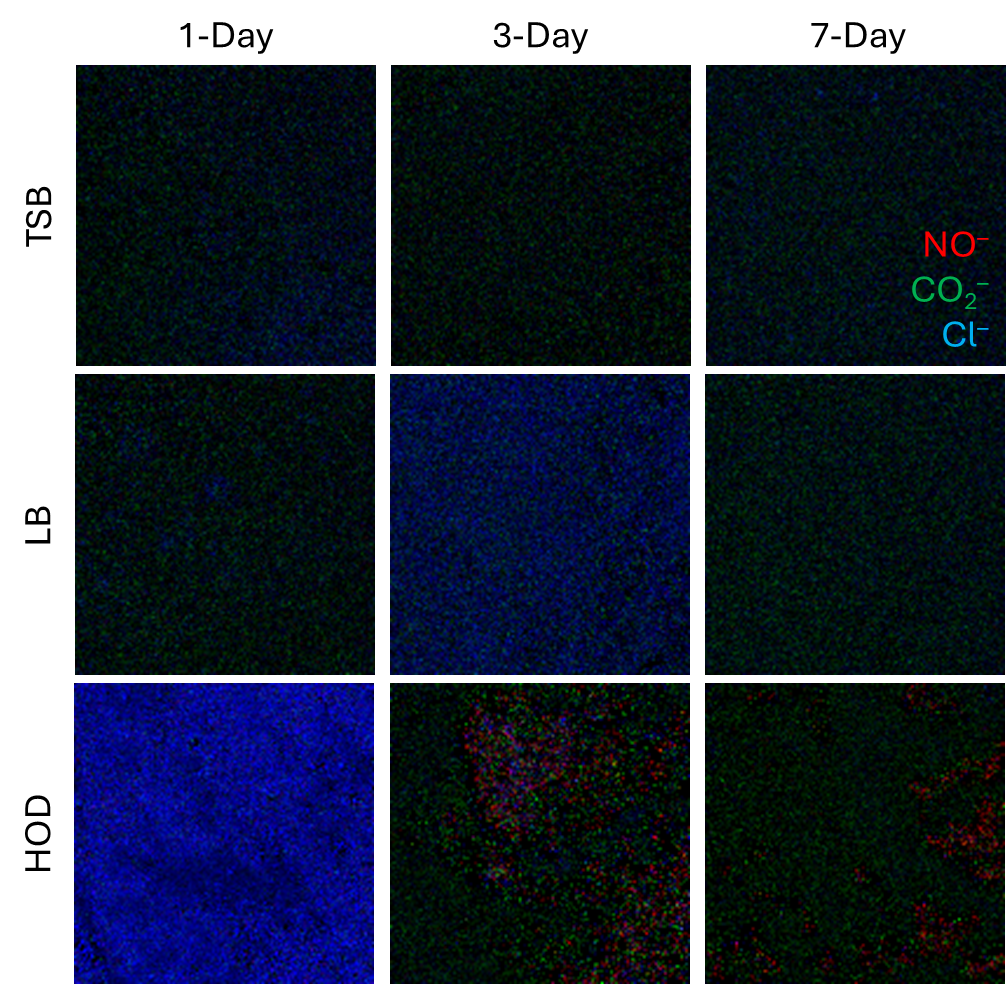


500 μm

Figure S13: RGB overlay of nitric oxide NO^–^(red), carbon dioxide CO_2_^–^(green), and chlorine Cl^–^(blue) with intensity values normalized to 0.2 total ion intensity.

**Figure S13** shows the red, green, and blue (RGB) overlay of the molecules in **Figure S12.** Chlorine is very prominent within the HOD media during the 1-day analysis. The scanning area is 500 μm by 500 μm.


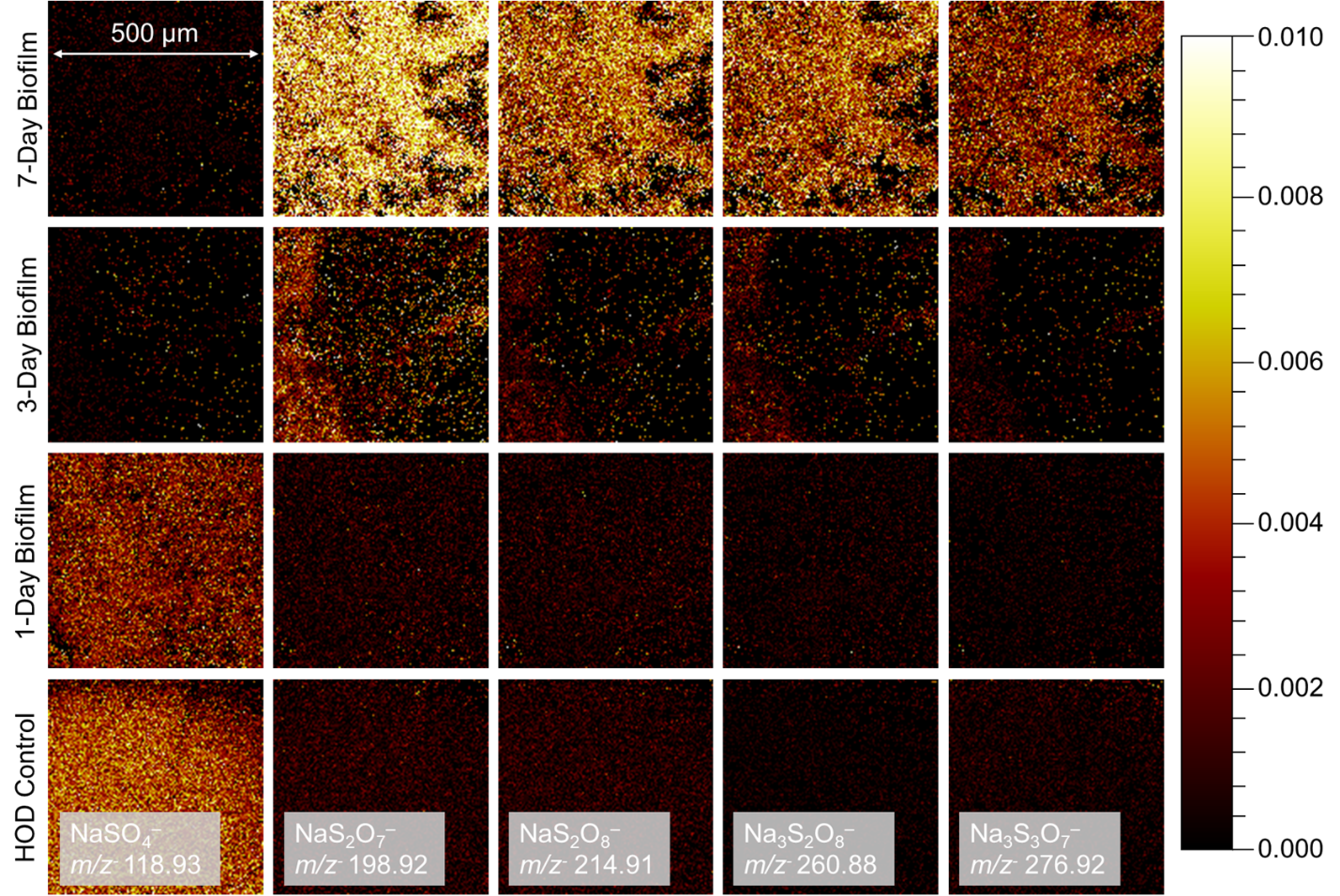


Figure S14: Normalized ToF-SIMS 2D images showing spatial distribution of sodium sulfate molecules observed within the spectra and increasing intensity at 7-Day growth correlating with cellular death.

**Figure S14** shows sodium sulfate molecules increasing in intensity at 7-Day biofilm growth which correlates to the growth curve indication of cellular death. The intensity values are normalized to total ion image for each of the sample sets. Increase in intensity can provide a link to cell stress and death.

# Supplementary Tables

Table S1: Hydrogen oxidizing de-nitrifier bacterial growth media constituent concentrations.

| **Constituent** | **Initial Concentration** | |
| --- | --- | --- |
|  | (mg/L) | (mM) |
| CaCl_2_•2H_2_O | 88.2 | 0.600 |
| MgCl_2_ | 47.6 | 0.500 |
| KCl | 14.9 | 0.200 |
| NH_4_Cl | 53.5 | 1.00 |
| NaH_2_PO_4_ | 12.0 | 0.100 |
| HEPES | 7149 | 30.0 |
| Wolfe’s vitamins^2^ | 1× | |
| Wolfe’s minerals^3^ | 1× | |
| glucose | 1,800 | 10.0 |
| ^1^Concentrations given are based on values of each constituent added (solution concentrations could be lower due to precipitation or biomass uptake). | | |
| ^2^ATCC® MDVS™ | | |
| ^3^ATCC® MDTMS™ | | |

Table S2: Concentrations of Wolfe’s vitamins and minerals included in the hydrogen oxidizing de-nitrifier bacterial growth media.

| **Wolfe’s Vitamins** | | | **Wolfe’s Minerals** | | |
| --- | --- | --- | --- | --- | --- |
| Constituent | Concentration | | Constituent | Concentration | |
|  | (µg/L) | (µM) |  | (mg/L) | (µM) |
| Folic Acid | 20.0 | 0.0453 | EDTA | 5.00 | 17.1 |
| Pyridoxine Hydrochloride | 100 | 0.486 | MgSO_4_ · 7H_2_O | 30.0 | 122 |
| Riboflavin | 50.0 | 0.133 | MnSO_4_ · H2O | 5.00 | 29.6 |
| Biotin | 20.0 | 0.0819 | NaCl | 10.0 | 171 |
| Thiamine | 50.0 | 0.166 | FeSO_4_ · 7H_2_O | 1.00 | 3.60 |
| Nicotinic Acid | 50.0 | 0.406 | Co(NO_3_)_2_ · 6H_2_O | 1.00 | 3.44 |
| Calcium Pantothenate | 50.0 | 0.210 | CaCl_2_ (anhydrous) | 1.00 | 9.01 |
| Vitamin B12 | 1.00 | 0.000738 | ZnSO_4_ · 7H_2_O | 1.00 | 3.48 |
| P-aminobenzoic Acid | 50.0 | 0.365 | CuSO_4_ · 5H_2_O | 0.100 | 0.400 |
| Thioctic Acid | 50.0 | 0.242 | AlK(SO_4_)_2_ (anhydrous) | 0.100 | 0.387 |
| Monopotassium Phosphate | 9,000 | 66.1 | H_3_BO_3_ | 0.100 | 1.62 |
|  |  |  | Na_2_MoO_4_ · 2H_2_O | 0.100 | 0.413 |
|  |  |  | Na_2_SeO_3_ (anhydrous) | 0.0100 | 0.0578 |
|  |  |  | Na_2_WO_4_ · 2H_2_O | 0.100 | 0.303 |
|  |  |  | NiCl_2_ · 6H_2_O | 0.200 | 0.842 |

Table S3: Tryptic soy broth and LB broth media composition.

| **Tryptic Soy Broth** | |
| --- | --- |
| Constituent | Initial Concentration (mg/L) |
| Cesin peptone (pancreatic) | 17000 |
| Sodium chloride | 5000 |
| Soya peptone (papain digest) | 3000 |
| Dipotassium phosphate | 2500 |
| Glucose | 2500 |
| Final pH: 7.3 ± 0.2 at 25 °C  Sigma-Aldrich 22092-500G  Lot: BCR0572 | |
| **LB Broth** | |
| Constituent | Initial Concentration (mg/L) |
| Cesin peptone | 10000 |
| Yeast Extract | 5000 |
| Sodium chloride | 5000 |
| Fisher Bioreagents BP9722-500  Lot: 135062 | |

## Table S4: Peak Reduction for Media Related Prominent Peaks.

| **1-Day Biofilm Growth in HOD Medium** | | | | |
| --- | --- | --- | --- | --- |
| *m/z^–^ _theo._* | *m/z^–^_obs._* | *Peak Area* | *Peak Area Reduction %* | *Abs. Peak Area Reduction %* |
| 106.99 | 106.98 | 298081.86 | -55.9 | 55.9 |
| 136.01 | 136.01 | 120819.21 | -65.2 | 65.2 |
| 237.09 | 237.10 | 137622.21 | -73.5 | 73.5 |
| 340.24 | 340.07 | 3476.54 | -83.2 | 83.2 |
| 497.26 | 497.23 | 2325.95 | -92.4 | 92.4 |
| **3-Day Biofilm Growth in HOD Medium** | | | | |
| *m/z^–^ _theo._* | *m/z^–^_obs._* | *Peak Area* | *Peak Area Reduction %* | *Abs. Peak Area Reduction %* |
| 106.99 | 106.97 | 9315.69 | -98.6 | 98.6 |
| 136.01 | 136.01 | 4180.38 | -98.7 | 98.7 |
| 237.09 | 237.12 | 2039.37 | -99.6 | 99.6 |
| 340.24 | 340.19 | 524.31 | -97.4 | 97.4 |
| 497.26 | 497.27 | 149.03 | -99.5 | 99.5 |
| **7-Day Biofilm Growth in HOD Medium** | | | | |
| *m/z^–^ _theo._* | *m/z^–^_obs._* | *Peak Area* | *Peak Area Reduction %* | *Abs. Peak Area Reduction %* |
| 106.99 | 106.99 | 9782.72 | -98.5 | 98.5 |
| 136.01 | 136.00 | 8590.71 | -97.5 | 97.5 |
| 237.09 | 237.10 | 3933.52 | -99.2 | 99.2 |
| 340.24 | 340.14 | 920.99 | -95.5 | 95.5 |
| 497.26 | 497.25 | 521.35 | -98.3 | 98.3 |
| **HOD Medium Control** | | | | |
| *m/z^–^ _theo._* | *m/z^–^_obs._* | *Peak Area* |  |  |
| 106.99 | 106.98 | 676165.86 |  |  |
| 136.01 | 136.01 | 347610.2 |  |  |
| 237.09 | 237.11 | 519476.56 |  |  |
| 340.24 | 340.07 | 20759.8 |  |  |
| 497.26 | 497.22 | 30718.88 |  |  |

Table S5: Peak identifications for 1-day, 3-day, and 7-day growth for HOD, LB, and TSB growth media in the mass range of *m/z^–^* 200 – 350.

|  |  |  |  |  |  | HOD | | | LB | | | TSB | | |
| --- | --- | --- | --- | --- | --- | --- | --- | --- | --- | --- | --- | --- | --- | --- |
| m/z^−^_theo._ | m/z^−^_obs._ | ∆M, ppm | Species | Fragment | Assignment | 24 Hrs. | 3 Days | 7 Days | 24 Hrs. | 3 Days | 7 Days | 24 Hrs. | 3 Days | 7 Days |
| 203.04622 | 203.046698 | 2.374154 | $C_{10}H_{7}N_{2}O_{3}^{-}$ | [M-H] | Analine | x |  |  |  |  |  |  |  |  |
| 205.06589 | 205.0638 | -10.184342 | $C_{15}H_{9}O^{-}$ | [M-H] | Anthraldehyde | x |  |  |  |  |  |  |  |  |
| 207.06896 | 207.071648 | 12.969053 | $C_{14}H_{9}NO^{-}$ | [M-H] | Oxidoanthracen amine | x |  |  |  |  |  |  |  |  |
| 209.02442 | 209.027946 | 16.88248 | $C_{13}H_{5}O_{3}^{-}$ | [M-H] | Phenalene Trione |  |  |  |  | x | x |  | x | x |
| 209.07791 | 209.074138 | -18.038792 | $C_{6}H_{13}N_{2}O_{6}^{-}$ | [M-H] | Diaminoglucose | x |  |  |  |  |  |  |  |  |
| 211.04007 | 211.042469 | 11.38014 | $C_{13}H_{7}O_{3}^{-}$ | [M-H] | Hydroxyanthone |  | x | x | x | x | x | x | x | x |
| 211.17035 | 211.172097 | 8.255911 | $C_{13}H_{23}O_{2}^{-}$ | [M-H] | Trideconic acid |  | x | x |  |  |  |  |  |  |
| 217.06187 | 217.06815 | 28.950819 | $C_{11}H_{9}N_{2}O_{3}^{-}$ | [M-5H] | Phenylalanylglycine | x |  |  |  |  |  |  |  |  |
| 219.07752 | 219.082767 | 23.967444 | $C_{11}H_{11}N_{2}O_{3}^{-}$ | [M-3H] | Phenylalanylglycine | x |  |  |  |  |  |  |  |  |
| 220.08534 | 220.085731 | 1.771135 | $C_{11}H_{12}N_{2}O_{3}^{-}$ | [M-2H] | Phenylalanylglycine | x |  |  |  |  |  |  |  |  |
| 221.09317 | 221.082701 | -47.330993 | $C_{11}H_{13}N_{2}O_{3}^{-}$ | [M-H] | Phenylalanylglycine | x |  |  |  |  |  |  |  |  |
| 223.02166 | 223.033711 | 54.04743 | $C_{6}H_{11}N_{2}O_{3}S_{2}^{-}$ | [M-H] | Cysteylcysteine |  |  |  |  |  | x | x | x | x |
| 225.05572 | 225.051162 | -20.244327 | $C_{14}H_{9}O_{3}^{-}$ | [M-H] | Bezoic Anhydride |  |  |  |  | x | x | x | x | x |
| 225.186 | 225.189725 | 16.525768 | $C_{14}H_{25}O_{2}^{-}$ | [M-3H] | Myristic Acid | x | x | x | x | x | x |  |  |  |
| 227.20165 | 227.207733 | 26.759387 | $C_{14}H_{27}O_{2}^{-}$ | [M-H] | Myristic Acid | x | x | x | x | x | x | x | x | x |
| 228.20948 | 228.205576 | -17.102417 | $C_{14}H_{28}O_{2}^{-}$ | [M] | Myristic Acid |  | x | x | x | x | x | x | x | x |
| 233.07791 | 233.076196 | -7.352483 | $C_{8}H_{13}N_{2}O_{6}^{-}$ | [M-H] | Dipeptide | x |  |  |  |  |  |  |  |  |
| 235.0758 | 235.08276 | 29.601777 | $C_{8}H_{15}SN_{2}O_{4}^{-}$ | [M-3H] | HEPES | x |  |  |  |  |  |  |  |  |
| 236.08363 | 236.093684 | 42.600568 | $C_{8}H_{16}SN_{2}O_{4}^{-}$ | [M-2H] | HEPES | x |  |  |  |  |  |  |  |  |
| 237.09145 | 237.099941 | 35.809537 | $C_{8}H_{17}SN_{2}O_{4}^{-}$ | [M-H] | HEPES | x | x | x |  | x |  |  | x | x |
| 238.09928 | 238.103196 | 16.460596 | $C_{8}H_{18}SN_{2}O_{4}^{-}$ | [M] | HEPES | x |  |  |  |  |  |  | x | x |
| 239.1071 | 239.101448 | -23.643233 | $C_{8}H_{19}SN_{2}O_{4}^{-}$ | [M+H] | HEPES | x |  |  |  |  |  | x | x | x |
| 239.20165 | 239.20423 | 10.769658 | $C_{15}H_{27}O_{2}^{-}$ | [M-3H] | Pentadecanoic Acid |  | x | x | x | x | x |  |  |  |
| 240.07919 | 240.081771 | 10.73964 | $C_{15}H_{12}O_{3}^{-}$ | [M] | Hydroxyflavone |  |  |  |  |  |  | x | x | x |
| 241.2173 | 241.219864 | 10.6141 | $C_{15}H_{29}O_{2}^{-}$ | [M-H] | Pentadecanoic Acid | x | x | x | x | x | x | x | x | x |
| 242.22513 | 242.223689 | -5.944663 | $C_{15}H_{30}O_{2}^{-}$ | [M] | Pentadecanoic Acid |  | x | x | x | x | x | x | x | x |
| 251.07137 | 251.075179 | 15.179352 | $C_{16}H_{11}O_{3}^{-}$ | [M-3H] | Chalepensin | x |  |  |  |  |  |  | x | x |
| 253.08702 | 253.089471 | 9.693574 | $C_{16}H_{13}O_{3}^{-}$ | [M-H] | Chalepensin |  |  |  |  |  |  | x | x | x |
| 253.2173 | 253.219133 | 7.225276 | $C_{16}H_{29}O_{2}^{-}$ | [M-3H] | Palmitic Acid | x | x | x | x | x | x |  |  |  |
| 255.23295 | 255.23487 | 7.508138 | $C_{16}H_{31}O_{2}^{-}$ | [M-H] | Palmitic Acid | x | x | x | x | x | x | x | x | x |
| 256.24078 | 256.238995 | -6.960165 | $C_{16}H_{32}O_{2}^{-}$ | [M] | Palmitic Acid | x | x | x | x | x | x | x | x | x |
| 259.09356 | 259.077711 | -61.170498 | $C_{10}H_{15}N_{2}O_{6}^{-}$ | [M-H] | Glutamylhydroxyproline | x |  |  |  |  |  |  |  |  |
| 267.07349 | 267.069927 | -13.351289 | $C_{10}H_{11}N_{4}O_{5}^{-}$ | [M-H] | Inosine | x |  |  |  |  |  |  |  |  |
| 267.10267 | 267.104229 | 5.846337 | $C_{17}H_{15}O_{3}^{-}$ | [M-H] | Dimethoxychalcone |  |  |  |  | x | x | x | x | x |
| 269.2486 | 269.252024 | 12.703109 | $C_{17}H_{33}O_{2}^{-}$ | [M-H] | Heptadecanoic Acid | x | x | x | x | x | x | x | x | x |
| 275.16527 | 275.176516 | 40.876251 | $C_{17}H_{23}O_{3}^{-}$ | [M-H] | Shogaol |  |  |  |  |  |  | x | x | x |
| 281.11832 | 281.123295 | 17.703237 | $C_{18}H_{17}O_{3}^{-}$ | [M-H] | Isoeugenol Phenylacetate |  |  |  |  | x | x | x | x | x |
| 281.2486 | 281.250749 | 7.625553 | $C_{18}H_{33}O_{2}^{-}$ | [M-3H] | Stearic Acid | x | x | x | x | x | x |  |  |  |
| 282.25643 | 282.254235 | -7.772594 | $C_{18}H_{34}O_{2}^{-}$ | [M-2H] | Stearic Acid | x | x |  |  |  | x |  |  |  |
| 283.26425 | 283.265459 | 4.253915 | $C_{18}H_{35}O_{2}^{-}$ | [M-H] | Stearic Acid | x | x | x | x | x | x | x | x | x |
| 284.27208 | 284.268768 | -11.648689 | $C_{18}H_{36}O_{2}^{-}$ | [M] | Stearic Acid | x | x | x |  | x | x | x | x | x |
| 285.2799 | 285.272925 | -24.709733 | $C_{18}H_{37}O_{2}^{-}$ | [M+H] | Stearic Acid |  | x | x |  | x | x |  | x | x |
| 296.14179 | 296.148118 | 21.358906 | $C_{19}H_{20}O_{3}^{-}$ | [M-4H] | Sterol Lipid |  |  |  |  |  |  |  | x | x |
| 295.13397 | 295.143475 | 32.211032 | $C_{19}H_{19}O_{3}^{-}$ | [M-5H] | Sterol Lipid |  |  |  |  | x | x | x | x | x |
| 297.14962 | 297.159322 | 32.656963 | $C_{19}H_{21}O_{3}^{-}$ | [M-3H] | Sterol Lipid |  |  |  |  | x | x | x | x | x |
| 298.15744 | 298.161073 | 12.17538 | $C_{19}H_{22}O_{3}^{-}$ | [M-2H] | Sterol Lipid |  |  |  |  | x | x | x | x | x |
| 299.16527 | 299.157518 | -25.90448 | $C_{19}H_{23}O_{3}^{-}$ | [M-H] | Sterol Lipid |  |  |  |  | x |  | x | x | x |
| 309.14962 | 309.160381 | 34.814832 | $C_{20}H_{21}O_{3}^{-}$ | [M-3H] | Rubifolide |  |  |  |  | x | x | x | x | x |
| 310.15744 | 310.164384 | 22.380004 | $C_{20}H_{22}O_{3}^{-}$ | [M-2H] | Rubifolide |  |  |  |  | x | x | x | x | x |
| 311.16527 | 311.176722 | 36.808633 | $C_{20}H_{23}O_{3}^{-}$ | [M-H] | Rubifolide |  |  |  | x | x | x | x | x | x |
| 311.29555 | 311.295262 | -0.936978 | $C_{20}H_{39}O_{2}^{-}$ | [M-H] | Arachidic Acid | x | x | x |  |  |  |  |  |  |
| 312.17309 | 312.175912 | 9.028391 | $C_{20}H_{24}O_{3}^{-}$ | [M] | Rubifolide |  | x |  | x | x | x | x | x | x |
| 313.18092 | 313.171486 | -30.117407 | $C_{20}H_{25}O_{3}^{-}$ | [M+H] | Rubifolide | x |  |  |  | x | x | x | x | x |
| 323.16527 | 323.175239 | 30.85269 | $C_{21}H_{23}O_{3}^{-}$ | [M-3H] | Sterol Lipid |  |  |  |  | x | x | x | x | x |
| 325.18092 | 325.192396 | 35.297392 | $C_{21}H_{25}O_{3}^{-}$ | [M-H] | Sterol Lipid |  | x |  | x | x | x | x | x | x |
| 326.18874 | 326.191365 | 8.037142 | $C_{21}H_{26}O_{3}^{-}$ | [M] | Sterol Lipid |  |  |  | x | x | x | x | x | x |
| 327.19657 | 327.186605 | -30.451185 | $C_{21}H_{27}O_{3}^{-}$ | [M-H] | Sterol Lipid |  |  |  | x | x | x | x | x | x |
| 328.20439 | 328.188805 | -47.496131 | $C_{21}H_{28}O_{3}^{-}$ | [M] | Sterol Lipid |  |  |  |  | x | x | x | x | x |
| 337.18092 | 337.191343 | 30.918717 | $C_{22}H_{25}O_{3}^{-}$ | [M-3H] | Sterol Lipid |  |  |  |  | x | x | x | x | x |
| 339.19657 | 339.207971 | 33.6176 | $C_{22}H_{27}O_{3}^{-}$ | [M-H] | Sterol Lipid |  | x | x | x | x | x | x | x | x |
| 340.20439 | 340.207411 | 8.870113 | $C_{22}H_{28}O_{3}^{-}$ | [M] | Sterol Lipid |  |  |  | x | x | x | x | x | x |
| 341.21222 | 341.231491 | 56.48384 | $C_{22}H_{29}O_{3}^{-}$ | [M+H] | Sterol Lipid |  |  |  | x | x | x | x | x | x |
| Note: *m/z*^−^ represents the mass-to-charge ratio for negatively charged ions  *m/z*^−^_theo._ represents the theoretical mass to charge ratio  *m/z*^−^_obs._ represents the observed mass to charge ratio  ΔM, ppm is the mass deviation recorded for the observed *m/z*^−^ against the theoretical *m/z*^−^ in parts per million (Yu et al., 2023). | | | | | | | | | | | | | | |

Table S6: Signal to noise ratio (SNR) values for peak identifications of 1-day, 3-day, and 7-day growth using HOD, LB, and TSB growth media in the mass range of *m/z^–^* 200 – 350.

|  |  |  |  |  |  | HOD SNR | | | LB SNR | | | TSB SNR | | |
| --- | --- | --- | --- | --- | --- | --- | --- | --- | --- | --- | --- | --- | --- | --- |
| m/z^−^_theo._ | m/z^−^_obs._ | ∆M, ppm | Species | Fragment | Assignment | 24 Hrs. | 3 Days | 7 Days | 24 Hrs. | 3 Days | 7 Days | 24 Hrs. | 3 Days | 7 Days |
| 203.04622 | 203.046698 | 2.374154 | $C_{10}H_{7}N_{2}O_{3}^{-}$ | [M-H] | Analine | 11.60 |  |  |  |  |  |  |  |  |
| 205.06589 | 205.0638 | -10.184342 | $C_{15}H_{9}O^{-}$ | [M-H] | Anthraldehyde | 16.04 |  |  |  |  |  |  |  |  |
| 207.06896 | 207.071648 | 12.969053 | $C_{14}H_{9}NO^{-}$ | [M-H] | Oxidoanthracen amine | 10.40 |  |  |  |  |  |  |  |  |
| 209.02442 | 209.027946 | 16.88248 | $C_{13}H_{5}O_{3}^{-}$ | [M-H] | Phenalene Trione |  |  |  |  | 11.96 | 10.40 |  | 24.07 | 29.38 |
| 209.07791 | 209.074138 | -18.038792 | $C_{6}H_{13}N_{2}O_{6}^{-}$ | [M-H] | Diaminoglucose | 5.41 |  |  |  |  |  |  |  |  |
| 211.04007 | 211.042469 | 11.38014 | $C_{13}H_{7}O_{3}^{-}$ | [M-H] | Hydroxyanthone |  | 8.68 | 10.41 | 23.40 | 17.16 | 18.62 | 18.51 | 25.69 | 33.15 |
| 211.17035 | 211.172097 | 8.255911 | $C_{13}H_{23}O_{2}^{-}$ | [M-H] | Trideconic acid |  | 7.15 | 7.94 |  |  |  |  |  |  |
| 217.06187 | 217.06815 | 28.950819 | $C_{11}H_{9}N_{2}O_{3}^{-}$ | [M-5H] | Phenylalanylglycine | 14.30 |  |  |  |  |  |  |  |  |
| 219.07752 | 219.082767 | 23.967444 | $C_{11}H_{11}N_{2}O_{3}^{-}$ | [M-3H] | Phenylalanylglycine | 23.81 |  |  |  |  |  |  |  |  |
| 220.08534 | 220.085731 | 1.771135 | $C_{11}H_{12}N_{2}O_{3}^{-}$ | [M-2H] | Phenylalanylglycine | 6.15 |  |  |  |  |  |  |  |  |
| 221.09317 | 221.082701 | -47.330993 | $C_{11}H_{13}N_{2}O_{3}^{-}$ | [M-H] | Phenylalanylglycine | 17.38 |  |  |  |  |  |  |  |  |
| 223.02166 | 223.033711 | 54.04743 | $C_{6}H_{11}N_{2}O_{3}S_{2}^{-}$ | [M-H] | Cysteylcysteine |  |  |  |  |  | 5.48 | 6.34 | 35.13 | 26.22 |
| 225.05572 | 225.051162 | -20.244327 | $C_{14}H_{9}O_{3}^{-}$ | [M-H] | Bezoic Anhydride |  |  |  |  | 7.56 | 5.96 | 10.46 | 23.80 | 29.75 |
| 225.186 | 225.189725 | 16.525768 | $C_{14}H_{25}O_{2}^{-}$ | [M-3H] | Myristic Acid | 4.43 | 10.14 | 10.95 | 7.96 | 8.99 | 9.85 |  |  |  |
| 227.20165 | 227.207733 | 26.759387 | $C_{14}H_{27}O_{2}^{-}$ | [M-H] | Myristic Acid | 4.86 | 19.24 | 33.68 | 35.12 | 46.60 | 47.90 | 29.43 | 18.07 | 19.24 |
| 228.20948 | 228.205576 | -17.102417 | $C_{14}H_{28}O_{2}^{-}$ | [M] | Myristic Acid |  | 6.61 | 12.04 | 12.13 | 17.25 | 16.49 | 10.20 | 5.08 | 5.37 |
| 233.07791 | 233.076196 | -7.352483 | $C_{8}H_{13}N_{2}O_{6}^{-}$ | [M-H] | Dipeptide | 9.16 |  |  |  |  |  |  |  |  |
| 235.0758 | 235.08276 | 29.601777 | $C_{8}H_{15}SN_{2}O_{4}^{-}$ | [M-3H] | HEPES | 29.53 |  |  |  |  |  |  |  |  |
| 236.08363 | 236.093684 | 42.600568 | $C_{8}H_{16}SN_{2}O_{4}^{-}$ | [M-2H] | HEPES | 8.53 |  |  |  |  |  |  |  |  |
| 237.09145 | 237.099941 | 35.809537 | $C_{8}H_{17}SN_{2}O_{4}^{-}$ | [M-H] | HEPES | 57.49 | 6.26 | 7.72 |  | 6.88 |  |  | 19.44 | 15.98 |
| 238.09928 | 238.103196 | 16.460596 | $C_{8}H_{18}SN_{2}O_{4}^{-}$ | [M] | HEPES | 18.38 |  |  |  |  |  |  | 8.73 | 7.01 |
| 239.1071 | 239.101448 | -23.643233 | $C_{8}H_{19}SN_{2}O_{4}^{-}$ | [M+H] | HEPES | 11.98 |  |  |  |  |  | 13.84 | 26.49 | 30.14 |
| 239.20165 | 239.20423 | 10.769658 | $C_{15}H_{27}O_{2}^{-}$ | [M-3H] | Pentadecanoic Acid |  | 10.57 | 13.94 | 11.91 | 11.94 | 12.91 |  |  |  |
| 240.07919 | 240.081771 | 10.73964 | $C_{15}H_{12}O_{3}^{-}$ | [M] | Hydroxyflavone |  |  |  |  |  |  | 4.53 | 9.86 | 11.59 |
| 241.2173 | 241.219864 | 10.6141 | $C_{15}H_{29}O_{2}^{-}$ | [M-H] | Pentadecanoic Acid | 3.55 | 45.03 | 73.75 | 63.74 | 79.26 | 80.15 | 61.69 | 40.87 | 56.97 |
| 242.22513 | 242.223689 | -5.944663 | $C_{15}H_{30}O_{2}^{-}$ | [M] | Pentadecanoic Acid |  | 18.27 | 31.05 | 25.75 | 32.71 | 32.70 | 25.25 | 15.41 | 22.00 |
| 251.07137 | 251.075179 | 15.179352 | $C_{16}H_{11}O_{3}^{-}$ | [M-3H] | Chalepensin | 11.43 |  |  |  |  |  |  | 6.35 | 8.12 |
| 253.08702 | 253.089471 | 9.693574 | $C_{16}H_{13}O_{3}^{-}$ | [M-H] | Chalepensin |  |  |  |  |  |  | 12.94 | 18.12 | 24.54 |
| 253.2173 | 253.219133 | 7.225276 | $C_{16}H_{29}O_{2}^{-}$ | [M-3H] | Palmitic Acid | 11.94 | 17.09 | 15.04 | 16.17 | 14.87 | 17.17 |  |  |  |
| 255.23295 | 255.23487 | 7.508138 | $C_{16}H_{31}O_{2}^{-}$ | [M-H] | Palmitic Acid | 49.51 | 64.97 | 59.25 | 46.52 | 71.31 | 78.11 | 51.07 | 49.83 | 58.67 |
| 256.24078 | 256.238995 | -6.960165 | $C_{16}H_{32}O_{2}^{-}$ | [M] | Palmitic Acid | 18.64 | 27.94 | 24.78 | 18.62 | 29.95 | 32.91 | 20.98 | 20.26 | 24.08 |
| 259.09356 | 259.077711 | -61.170498 | $C_{10}H_{15}N_{2}O_{6}^{-}$ | [M-H] | Glutamylhydroxyproline | 15.93 |  |  |  |  |  |  |  |  |
| 267.07349 | 267.069927 | -13.351289 | $C_{10}H_{11}N_{4}O_{5}^{-}$ | [M-H] | Inosine | 9.40 |  |  |  |  |  |  |  |  |
| 267.10267 | 267.104229 | 5.846337 | $C_{17}H_{15}O_{3}^{-}$ | [M-H] | Dimethoxychalcone |  |  |  |  | 7.81 | 6.74 | 12.35 | 19.95 | 25.96 |
| 269.2486 | 269.252024 | 12.703109 | $C_{17}H_{33}O_{2}^{-}$ | [M-H] | Heptadecanoic Acid | 6.28 | 15.29 | 17.02 | 8.92 | 13.75 | 15.71 | 11.01 | 7.17 | 10.16 |
| 275.16527 | 275.176516 | 40.876251 | $C_{17}H_{23}O_{3}^{-}$ | [M-H] | Shogaol |  |  |  |  |  |  | 11.20 | 9.64 | 16.74 |
| 281.11832 | 281.123295 | 17.703237 | $C_{18}H_{17}O_{3}^{-}$ | [M-H] | Isoeugenol Phenylacetate |  |  |  |  | 4.47 | 3.18 | 11.03 | 16.18 | 20.10 |
| 281.2486 | 281.250749 | 7.625553 | $C_{18}H_{33}O_{2}^{-}$ | [M-3H] | Stearic Acid | 16.50 | 24.60 | 11.06 | 4.58 | 6.32 | 9.41 |  |  |  |
| 282.25643 | 282.254235 | -7.772594 | $C_{18}H_{34}O_{2}^{-}$ | [M-2H] | Stearic Acid | 6.06 | 11.37 |  |  |  | 5.17 |  |  |  |
| 283.26425 | 283.265459 | 4.253915 | $C_{18}H_{35}O_{2}^{-}$ | [M-H] | Stearic Acid | 58.04 | 92.74 | 54.29 | 16.72 | 48.91 | 66.58 | 28.80 | 47.74 | 44.62 |
| 284.27208 | 284.268768 | -11.648689 | $C_{18}H_{36}O_{2}^{-}$ | [M] | Stearic Acid | 24.94 | 46.02 | 24.00 |  | 20.83 | 28.54 | 10.18 | 20.53 | 18.49 |
| 285.2799 | 285.272925 | -24.709733 | $C_{18}H_{37}O_{2}^{-}$ | [M+H] | Stearic Acid |  | 15.32 | 6.36 |  | 5.12 | 7.76 |  | 5.02 | 4.49 |
| 296.14179 | 296.148118 | 21.358906 | $C_{19}H_{20}O_{3}^{-}$ | [M-4H] | Sterol Lipid |  |  |  |  |  |  |  | 7.61 | 10.52 |
| 295.13397 | 295.143475 | 32.211032 | $C_{19}H_{19}O_{3}^{-}$ | [M-5H] | Sterol Lipid |  |  |  |  | 5.18 | 4.29 | 10.26 | 19.68 | 26.02 |
| 297.14962 | 297.159322 | 32.656963 | $C_{19}H_{21}O_{3}^{-}$ | [M-3H] | Sterol Lipid |  |  |  |  | 19.50 | 16.33 | 41.39 | 50.86 | 89.22 |
| 298.15744 | 298.161073 | 12.17538 | $C_{19}H_{22}O_{3}^{-}$ | [M-2H] | Sterol Lipid |  |  |  |  | 4.35 | 4.01 | 13.79 | 20.38 | 38.04 |
| 299.16527 | 299.157518 | -25.90448 | $C_{19}H_{23}O_{3}^{-}$ | [M-H] | Sterol Lipid |  |  |  |  | 3.15 |  | 7.40 | 10.11 | 21.00 |
| 309.14962 | 309.160381 | 34.814832 | $C_{20}H_{21}O_{3}^{-}$ | [M-3H] | Rubifolide |  |  |  |  | 10.13 | 9.00 | 15.66 | 24.14 | 31.51 |
| 310.15744 | 310.164384 | 22.380004 | $C_{20}H_{22}O_{3}^{-}$ | [M-2H] | Rubifolide |  |  |  |  | 3.64 | 3.61 | 5.32 | 9.43 | 12.81 |
| 311.16527 | 311.176722 | 36.808633 | $C_{20}H_{23}O_{3}^{-}$ | [M-H] | Rubifolide |  |  |  | 12.47 | 42.40 | 38.57 | 75.81 | 93.30 | 126.03 |
| 311.29555 | 311.295262 | -0.936978 | $C_{20}H_{39}O_{2}^{-}$ | [M-H] | Arachidic Acid | 6.02 | 14.98 | 8.92 |  |  |  |  |  |  |
| 312.17309 | 312.175912 | 9.028391 | $C_{20}H_{24}O_{3}^{-}$ | [M] | Rubifolide |  | 6.80 |  | 4.08 | 17.46 | 15.74 | 33.49 | 42.87 | 59.35 |
| 313.18092 | 313.171486 | -30.117407 | $C_{20}H_{25}O_{3}^{-}$ | [M+H] | Rubifolide | 5.53 |  |  |  | 7.57 | 6.54 | 17.28 | 24.34 | 34.77 |
| 323.16527 | 323.175239 | 30.85269 | $C_{21}H_{23}O_{3}^{-}$ | [M-3H] | Sterol Lipid |  |  |  |  | 11.76 | 9.98 | 16.04 | 24.15 | 29.45 |
| 325.18092 | 325.192396 | 35.297392 | $C_{21}H_{25}O_{3}^{-}$ | [M-H] | Sterol Lipid |  | 6.44 |  | 22.24 | 63.75 | 59.17 | 84.36 | 103.02 | 124.32 |
| 326.18874 | 326.191365 | 8.037142 | $C_{21}H_{26}O_{3}^{-}$ | [M] | Sterol Lipid |  |  |  | 9.11 | 29.11 | 26.43 | 38.75 | 49.02 | 60.55 |
| 327.19657 | 327.186605 | -30.451185 | $C_{21}H_{27}O_{3}^{-}$ | [M-H] | Sterol Lipid |  |  |  | 4.47 | 15.77 | 14.94 | 22.17 | 28.71 | 36.37 |
| 328.20439 | 328.188805 | -47.496131 | $C_{21}H_{28}O_{3}^{-}$ | [M] | Sterol Lipid |  |  |  |  | 5.36 | 4.27 | 7.71 | 10.85 | 13.23 |
| 337.18092 | 337.191343 | 30.918717 | $C_{22}H_{25}O_{3}^{-}$ | [M-3H] | Sterol Lipid |  |  |  |  | 10.60 | 9.60 | 13.44 | 20.60 | 25.61 |
| 339.19657 | 339.207971 | 33.6176 | $C_{22}H_{27}O_{3}^{-}$ | [M-H] | Sterol Lipid |  | 5.99 | 3.20 | 17.73 | 56.75 | 53.70 | 70.17 | 91.61 | 109.10 |
| 340.20439 | 340.207411 | 8.870113 | $C_{22}H_{28}O_{3}^{-}$ | [M] | Sterol Lipid |  |  |  | 6.58 | 26.18 | 24.60 | 31.77 | 44.31 | 53.61 |
| 341.21222 | 341.231491 | 56.48384 | $C_{22}H_{29}O_{3}^{-}$ | [M+H] | Sterol Lipid |  |  |  | 3.44 | 14.41 | 13.18 | 17.59 | 25.96 | 31.16 |
| Note: *m/z*^−^ represents the mass-to-charge ratio for negatively charged ions  *m/z*^−^_theo._ represents the theoretical mass to charge ratio  *m/z*^−^_obs._ represents the observed mass to charge ratio  ΔM, ppm is the mass deviation recorded for the observed *m/z*^−^ against the theoretical *m/z*^−^ in parts per million (Yu et al., 2023).  SNR, signal-to-noise ratio values based on mean intensity valyes divided by the standard deviation of the background signal. SNR>3 are considered true peaks. | | | | | | | | | | | | | | |

# References

Atasoy, M., Bartkova, S., Çetecioğlu-Gürol, Z., Nuno, O'byrne, C., Pérez-Rodríguez, F., Possas, A., Scheler, O., Sedláková-Kaduková, J., Sinčák, M., Steiger, M., Ziv, C., and Lund, P.A. (2024). Methods for studying microbial acid stress responses: from molecules to populations. *FEMS Microbiology Reviews* 48.

Baumgarten, T., Sperling, S., Seifert, J., Bergen, M.V., Steiniger, F., Wick, L.Y., and Heipieper, H.J. (2012). Membrane Vesicle Formation as a Multiple-Stress Response Mechanism Enhances Pseudomonas putida DOT-T1E Cell Surface Hydrophobicity and Biofilm Formation. *Applied and Environmental Microbiology* 78**,** 6217-6224.

Ding, Y., Zhou, Y., Yao, J., Szymanski, C., Fredrickson, J., Shi, L., Cao, B., Zhu, Z., and Yu, X.Y. (2016). In Situ Molecular Imaging of the Biofilm and Its Matrix. *Anal Chem* 88**,** 11244-11252.

Liao, X., Wu, Z., Li, Y., Luo, J., and Su, C. (2018). Enhanced degradation of polycyclic aromatic hydrocarbons by indigenous microbes combined with chemical oxidation. *Chemosphere* 213**,** 551-558.

Nguyen, K., and Kumar, P. (2022). Morphological Phenotypes, Cell Division, and Gene Expression of Escherichia coli under High Concentration of Sodium Sulfate. *Microorganisms* 10**,** 274.

Uruén, C., Chopo-Escuin, G., Tommassen, J., Mainar-Jaime, R.C., and Arenas, J. (2020). Biofilms as Promoters of Bacterial Antibiotic Resistance and Tolerance. *Antibiotics (Basel)* 10.

Yu, X.-Y., Yang, C., Gao, J., Xiong, J., Sui, X., Zhong, L., Zhang, Y., and Son, J. (2023). Molecular detection of per- and polyfluoroalkyl substances in water using time-of-flight secondary ion mass spectrometry. *Frontiers in Chemistry* 11.

Zhang, J.T., Brown, J., Scurr, D.J., Bullen, A., Maclellan-Gibson, K., Williams, P., Alexander, M.R., Hardie, K.R., Gilmore, I.S., and Rakowska, P.D. (2020). Cryo-OrbiSIMS for 3D Molecular Imaging of a Bacterial Biofilm in Its Native State. *Analytical Chemistry* 92**,** 9008-9015.
